# Characterizing Endogenous Protein Complexes with Biological Mass Spectrometry

**DOI:** 10.1021/acs.chemrev.1c00217

**Published:** 2021-08-18

**Authors:** Rivkah Rogawski, Michal Sharon

**Affiliations:** Department of Biomolecular Sciences, Weizmann Institute of Science, Rehovot 7610001, Israel

## Abstract

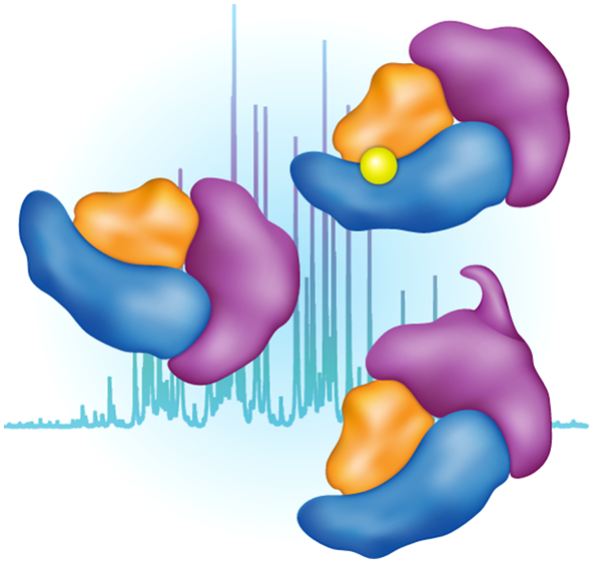

Biological mass spectrometry (MS) encompasses a range of methods for characterizing proteins and other biomolecules. MS is uniquely powerful for the structural analysis of endogenous protein complexes, which are often heterogeneous, poorly abundant, and refractive to characterization by other methods. Here, we focus on how biological MS can contribute to the study of endogenous protein complexes, which we define as complexes expressed in the physiological host and purified intact, as opposed to reconstituted complexes assembled from heterologously expressed components. Biological MS can yield information on complex stoichiometry, heterogeneity, topology, stability, activity, modes of regulation, and even structural dynamics. We begin with a review of methods for isolating endogenous complexes. We then describe the various biological MS approaches, focusing on the type of information that each method yields. We end with future directions and challenges for these MS-based methods.

## 1 Introduction

Proteins encoded by cellular DNA are the workhorses of the cell, carrying out diverse biochemical tasks that generate cellular phenotypes.^1^ However, many proteins do not act alone but associate with other proteins to form functional complexes, greatly increasing the complexity of molecular species found in the cell. Moreover, complex formation can depend on protein location and post-translational modifications (PTMs), further increasing the diversity of cellular species. A major challenge in biology is to map the cellular protein complexes and determine how complex composition varies as a function of cellular state. 22% of the protein coding genes in humans are represented in CORUM, a repository of experimentally studied complexes from mammalian organisms,^2^ and many more protein complexes likely exist.^3^ The topology and structural dynamics of protein complexes should be determined in the endogenous state, as similar to that as in cells or tissues, for the findings to have maximal physiological relevance. However, because of their limited quantity and heterogeneity, the range of biochemical and structural techniques that can be applied to endogenous complexes is restricted.

**Figure 1:**
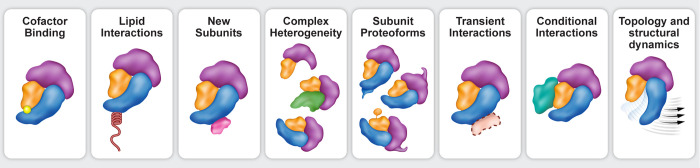
Biological MS yields a wide range of information about biological complexes. Depending on the specifics of sample preparation and the MS method chosen, biological MS can shed light on many properties of endogenous complexes. This includes determining binding of cofactors and lipids and their effects on complex composition, identification of new subunits, heterogeneity of complexes, post-translational modifications, and transient interactions or interactions that are only present under a distinct physiological condition. Structures and topologies, as well as structural dynamics, can also be revealed.

Biological mass spectrometry (MS), a term that encompasses a wide range of MS-based techniques, has provided tremendous insights into the composition and structural features of endogenous protein complexes. MS-based techniques balance throughput with resolution, filling a gap between time-intensive, all-atom structural techniques such as NMR and X-ray crystallography and high-throughput molecular biology techniques that do not provide detailed structural information. Here we detail the different MS based methods, focusing on the insights they provide into endogenous complexes and comparing between them. Depending on the approach taken, biological MS can determine the interaction partners for a particular protein, describe complex topology and structure, and even provide insights into conformational fluctuations (Figure 1).

This review is intended as an overview of sample preparation techniques and biological MS approaches available for in-depth characterization of endogenous complexes. Rather than focusing on technical aspects of specific MS techniques, which are detailed in many excellent reviews cited here^4−6^ and below, we focus on the compositional and structural insights that can be gained from biological MS and compare how different MS methods can yield this information. We also highlight recent exciting advances ranging from improvements in LC/MS technology to CRISPR/CAS9 technologies that enable epitope tagging of endogenous proteins, increasing relevance for a wider range of systems.

We begin by reviewing methods for isolating endogenous complexes, highlighting advantages and disadvantages of each. We define endogenous complexes as those composed of proteins expressed in the organism of origin, either under native promoters or under conditions as close to native promoters as possible. We then introduce the different biological MS methods for characterizing those complexes, pointing the reader to an updated technical review for each technology and detailing the advantages and disadvantages of each method. For each technology, we describe the biological information that it can provide, focusing on specific examples that highlight the power of biological MS and apologizing in advance for publications excluded due to lack of space. We end with a review of future directions and exciting developments that will open new frontiers in the study of protein complexes.

## 2 Preparation of Endogenous Protein Complexes for Mass Spectrometry

### 2.1 General Considerations for MS Protein Complex Sample Preparation

The information extracted from MS experiments is limited by the scale and quality of the input samples. Although many MS modalities can be applied to crude biological samples, as discussed below, typically assemblies must be extracted from cells or tissues and purified for MS analysis. It is crucial to remove as many contaminants, or copurifying proteins that are not part of the complex, as possible. In this section, we discuss general principles for the enrichment of protein complexes from different tissues and cell types. Once a suitable tissue and enrichment scheme has been selected, optimization of sample preparation protocol is a combination of art and science, and there is no substitute for screening many purification conditions. In fact, rapid screening platforms to test buffers and conditions in parallel, analogous to X-ray crystallography screens, have been used for MS analysis.^7^

**Figure 2:**
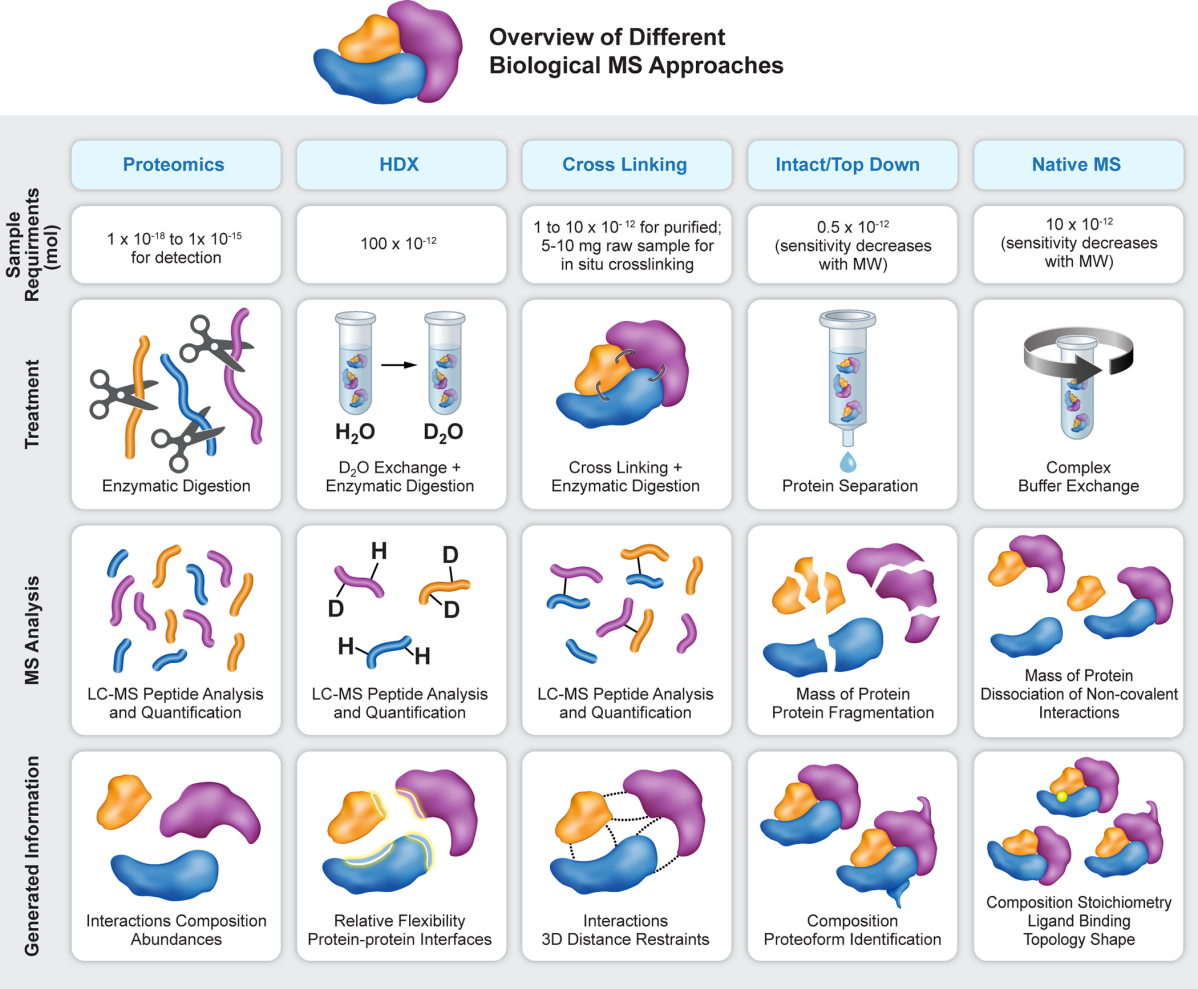
Overview of various biological MS techniques. Each biological MS method requires different amounts of protein (row 1: refs (162, 210, and 251) corroborate amounts for proteomic, in situ cross-linking, and HDX, respectively). For each method, the proteins must be treated in a different way (second row). While all include gas phase ion generation and MS analysis, the experimental setup differs slightly between samples, with bottom-up proteomic methods sequencing and quantifying peptides via LC/MS-MS and top-down and native MS measuring the mass of the whole protein and using tandem MS for protein fragmentation and dissociation of covalent and noncovalent interactions, respectively (third row). Each method yields different information, as detailed in section 3 and described in the last row.

Complex abundance will directly affect both the sample purification method as well as the choice of MS technique. Generally, bottom-up proteomics requires significantly less material than top-down MS methods (see section 3 and Figure 2). Therefore, while we focus primarily on purification strategies for proteins that are not overexpressed, we include a few strategies for ectopic overexpression with the caveat that overexpression might lead to nonphysiological complex formation. Moreover, for heteromeric complexes of unknown composition, not all subunits can be selected for overexpression. In some cases, overexpression of a single subunit may lead to proportionally altered levels of associated subunits such that subunit stoichiometry is preserved in the cell.^8^

**Figure 3:**
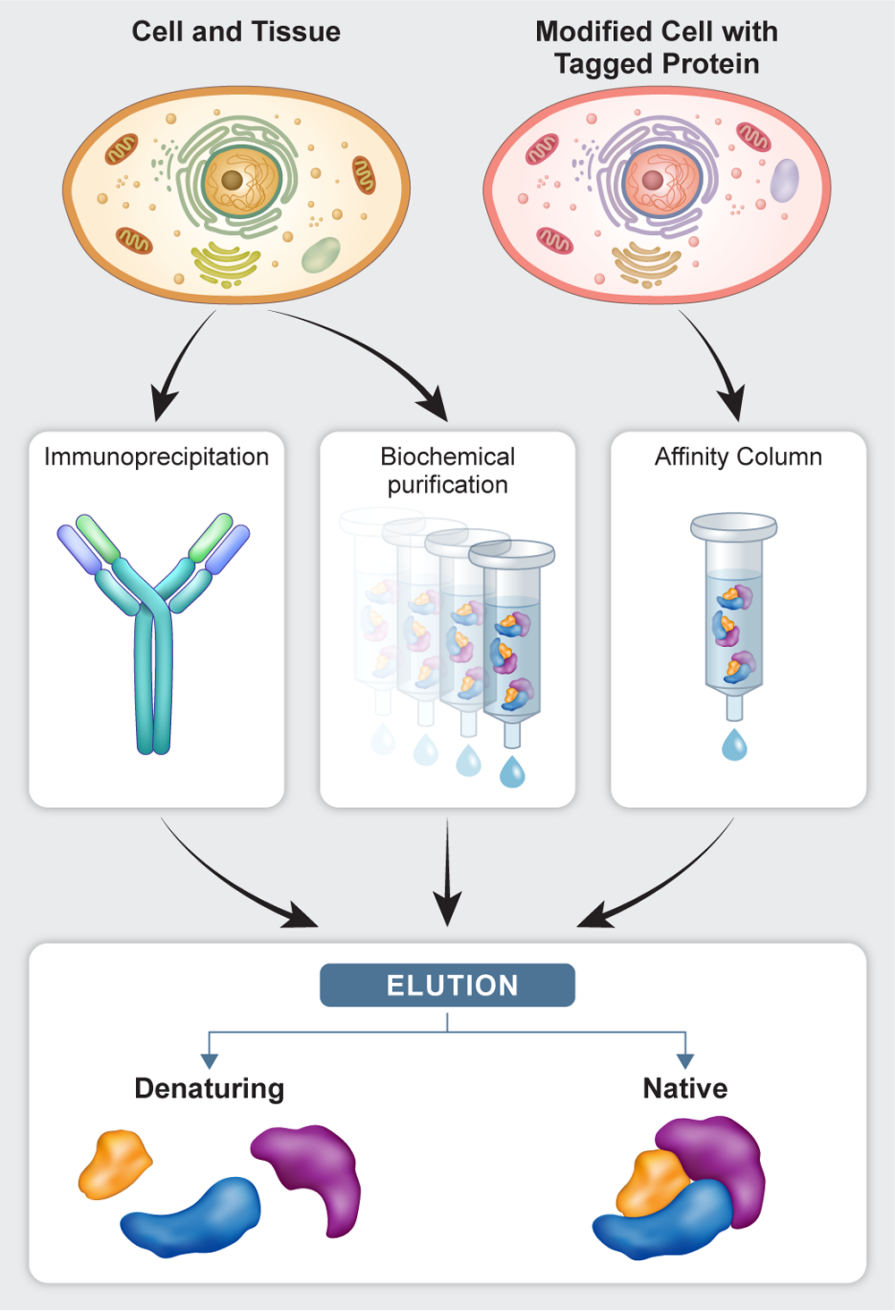
Strategies for protein complex isolation for biological MS. Purification of endogenous complexes can start either from unmodified cells and tissues, in which immunoprecipitation and biochemical purification can be applied, or by genetic manipulation of cells to express an epitope tagged protein, preferably at the endogenous locus (see section 2.4.1). At the end of the purification, complexes can either be eluted in a denatured state, in which case bottom-up proteomics or intact protein MS can be applied, or eluted in a native state, which will enable application of native-MS, HDX, or cross-linking MS.

Three main strategies exist for complex enrichment (Figure 3): biochemical purification, immunoprecipitation, and affinity tagging. All three strategies have yielded robust insight into biological complexes and their structural and compositional properties. For any strategy, it may be desirable to begin with subcellular fractionation to enable the retrieval of spatially defined complexes of interest from different cellular compartments such as the nuclei, cytosol, and mitochondria.^9,10^ Because compound assembly state can depend on cofactor binding, it can be important to include cofactors during purification to prevent complex disassembly. For example, the 26S proteasome dissociates readily once removed from cells if ATP is not included in the buffers in the physiological range of 1 mM.^11^ If the complex is known to have catalytic or enzymatic functions that can be reconstituted in vitro, it is worth developing in vitro assays that can be performed on fractions or purified complexes to validate that the complex is functional. For example, in our lab, 20S proteasome containing fractions are tested for degradation activity with fluorescent activity peptides.^12^

The desired final buffer and assembly state depend on the MS technique applied. For bottom-up proteomics analysis (see section 3.3.1), because proteins are digested and separated on a column, the complex does not need to be in an MS compatible buffer. In fact, the complex is typically denatured before digestion, and thus protein precipitation via organic solvents such as tricholoroacetic acid can be used to separate proteins from buffer components. After digestion, peptides are loaded onto a chromatography column that exchanges them into an MS compatible buffer. For chemical footprinting methods (see section 3.3.2), complexes should be in a native state for the foot-printing step but are then denatured and digested for MS analysis, relaxing complex purification requirements. However, buffers must be compatible with the chemistry of the label used. For example, for cross-linking MS with disuccinimidyl dibutyric urea, buffers containing primary amines should be avoided because they will react with the cross-linkers.^13^ For native and top-down MS (see section 3.4), complexes should be eluted in an assembled, native state and exchanged into MS compatible buffers, as detailed in ref (14). In particular, high concentrations of salts can suppress signal intensity and require rounds of buffer exchange. A number of salts that can maintain physiological ionic strength and pH while remaining compatible with MS, including ammonium acetate, ethylene diammonium diacetate buffer, and others.

### 2.2 Biochemical Purification

Biochemical purification strategies rely on differences in physicochemical properties between the complex of interest and its biological matrix. Starting from a homogenate of biological material, sequential centrifugation, precipitation, and/or chromatographic steps gradually yield solutions enriched in the complex of interest. Chromatographic purification steps can separate complexes based on their size (size exclusion chromatography), charge (ion exchange chromatography), or hydrophobicity (hydrophobic interaction chromatography). Protein complexes can also be enriched based on density via sucrose gradient centrifugation or via selective precipitation of some components, most popularly using ammonium sulfate. We refer the reader to guides for design of protein purification strategies for complete discussion of these techniques.^15^

The advantage of a biochemical purification is that it yields endogenous complexes that are unperturbed by the addition of non-native amino acid sequences or amplification of individual subunits. Complexes are also not bound to antibodies, which must be removed for downstream MS analysis. Disadvantages of biochemical purifications, and the reason that they have been primarily supplanted by affinity purification methodologies, is that they are lengthy, lossy, and must be optimized separately for each protein complex. The lengthiness can cause dissociation of transient interactions and the lossiness means that abundance of the complex must be quite high to provide enough starting material to survive the successive enrichment steps. Given these limitations, it is not surprising that biochemical purifications have been mainly applied to prepare stable and abundant complexes for MS analysis. These include the ribosome,^16^ the proteasome,^12^ the COP9 signalosome,^17^ and varying complexes of vinculun/Arp^18^ proteins.

Biochemical purification techniques are also used in cofractionation MS analysis of complexes, discussed below. In these experiments, complex biological samples are fractionated biochemically without tracking a particular component. Crude fractions are subjected to MS analysis, enabling identification of complexes either by proteomic colocalization or by directly observing the complex via native MS.^19^ A wide range of biochemical fractionation techniques have been used for this purpose, including ion exchange chromatgraphy,^19−21^ isoelectric focusing,^20−22^ sucrose density gradient centrifugation,^20,21^ size exclusion chromatography,^22,23^ native gel-based electrophoresis strategies,^24^ and capillary zone electrophoresis.^25^

### 2.3 Direct Immunoprecipitation

The ability to generate antibodies that bind specific epitopes in a protein of interest has accelerated many areas of biology. In protein purification, antibodies conjugated to sepharose or magnetic beads are used to immunoprecipitate (IP) protein complexes. Usually an antibody targeting one subunit in a complex is chosen, and IP under nondenaturing conditions will lead to isolation of the entire complex, provided that the epitope chosen is accessible in the complex. Reference (26) describes a protocol for choosing and optimizing a primary antibody for IP followed by MS analysis.

As with biochemical purifications, direct IP has the advantage that the protein does not need to be modified for purification. However, it requires robust antibodies that recognize the epitope. Antibodies can be conformer or proteoform specific, leading to a biased sampling of complex distribution. Typically, polyclonal antibodies are preferred over monoclonal antibodies because they can potentially recognize a range of epitopes and maintain population heterogeneity.

An additional consideration is dissociating the tightly bound protein/antibody complex after IP for MS analysis. This can be done by elution with high salt^27^ or via denaturing methods including low pH glycine buffer or urea. However, as discussed above, high salts and detergents are not compatible with all MS methods, and quaternary structure is destroyed by denaturing elution. Additionally, contamination of the purified sample with large quantities of the primary antibody can lead to masking of the MS signals of proteins of interest.

### 2.4 Affinity Tagging

Arguably, the most popular method for purifying endogenous protein complexes is affinity tagging, in which additional bases are added to the DNA sequence coding for the protein of interest. This sequence is transcribed along with the protein of interest, which then contains a tag that can be isolated using commercially available affinity resins for the tag of interest. Affinity methods provide a single-step purification in a high-throughput fashion that can be theoretically applied to any target of interest. The speed of purification can also preserve transient or weak biomolecular interactions.

A wide range of affinity tags are available, with novel tags introduced frequently (reviewed in refs (28−30)). Table 1 summarizes the properties of some popular tags for purification of endogenous complexes. Tandem affinity tags for tandem affinity purification (TAP) are also available that combine multiple epitopes for coupling multiple affinity steps.^31−34^ TAP approaches generally lead to better contaminant removal at the expense of dissociating transient interactions. The sensitivity of modern mass spectrometers combined with improved methods for contaminant detection makes the use of TAP tags less important.

**Table 1:** Properties of a Selection of Popular Tags for Affinity Purification Followed by MS Analysis

tag	nature	mass (kDa)	origin	elution method
HA, 3X HA	9 amino acids	1.102	human influenza hemagluttin^36^	low pH or HA peptide
C-myc	10 amino acids	1.202	C-myc gene product	low pH or myc peptide
GST	220 amino acids	26	enzyme glutathione-*S*-transferase	excess reduced glutathione
GFP	238 amino acids	27	green fluorescent protein	low pH or denaturing conditions
SpA	depends on how many repeats of IgG domain are used	1 engineered *Z*-domain: 6.6^37^	protein A from *Staphylococcus aureus*	SpA binding peptides, low pH, denaturing
FLAG, 3X-FLAG	8 amino acids	1.031	engineered^38^	FLAG-peptide, low pH, denaturing
CBP	26–28 amino acids	4	calmodulin binding peptide	EGTA and high salt
STREP	8 amino acids	1.058	genetic random library selection^39^	biotin or biotin derivative
His	6 amino acids	0.841	engineered^40^	imidazole or EDTA
V5	14 amino acids	1.421	simian virus 5	low pH or competitive elution with V5 peptide

When choosing a tag, its size, charge, and the method of removal from affinity resin is critical. As discussed above, this last property can be an extremely important variable for downstream MS analysis. If competitive elution strategies which involve addition of peptides or other small molecules interfere with downstream MS analysis because they limit the dynamic range, gel filtration or buffer exchange can reduce their concentration in the sample. Host organism is also an important consideration, with His tags being effective for purification of proteins from *E. coli* but significantly less useful from mammalian cells due to nonspecific copurification of contaminants.

Placement of the tag on the protein of interest is also crucial to avoid disrupting functional complex formation. Any known facts about protein structure and assembly should be incorporated. Tags can be placed at the N- or C-terminus of the protein, as well as internal to the sequence in a position accessible in the final folded protein structure.^35^ It is not possible to empirically predict which location is better for a protein of interest. For N-terminally tagged proteins, polypeptides that are improperly terminated early will contain the affinity tag and copurify along with the desired protein; for C-terminally tagged proteins, alternative initiation sites can also lead to a mixture of different proteins with the C-terminal tag. Ideally, biochemical or biological assays should confirm that the affinity tag has not impacted the protein’s endogenous fold, function, interactions, and localization. These can either be in vitro assays that assess enzymatic function or protein structural properties after tag addition, or *in cell* assays to confirm the location of the tagged protein and normal physiological state after tagging. However, it is always possible that the tag perturbs a completely unknown function or association that the protein engages in, and this should be considered as part of the experimental design and analysis.

#### 2.4.1 Methods for Generating Affinity Tagged Endogenous Complexes

To use affinity tags to purify endogenous complexes, the DNA coding for at least one subunit of the complex must be modified with the affinity tag. The gold standard, which presumably produces complexes as similar to native as possible, is to tag the protein at the endogenous gene locus in the appropriate host. Endogenous tagging strategies generally capitalize on the principle of homologous recombination, reviewed in refs (41 and 42,) in which homologous regions of DNA recombine and regions in between the homology patches can be swapped. Efficient homologous recombination relies on the generation of precise double-stranded DNA breaks (DSBs); the introduction of CRISPR/Cas9 technology,^43^ which revolutionized the simple and specific generation of DSBs, has advanced affinity tagging in a range of organisms, including *Escherichia coli*(44,45) and yeast.^46^ However, it is particularly powerful for endogenous tagging in mammalian cells, and several groups have presented tools and approaches for CRISPR based epitope tagging in mammalian cells.^47−50^ Moreover, the use of CRISPR to generate epitope tagged whole model organisms, such as mice,^51^ can significantly advance comparative purification of complexes from different tissues and under different physiological stresses. However, users of CRISPR should be aware that it can be subject to off-target effects.^52^

For *E. coli*,^41,53,54^ and yeast,^55−59^ robust tools for epitope tagging predate CRISPR/CAS9 technologies, and various libraries of epitope tagged yeast and *E. coli* strains are available, as well as yeast donor libraries that enable fast tagging of ORFs with any desired epitope.^60,61^ For mammalian cells, epitope tagging pre-CRISPR was generally accomplished via transcription activator-like effector nuclease (TALENS) or zinc finger nucleases(ZNFs),^48^ which are composed of protein domains that recognize specific DNA sequences fused to nonspecific DNA cleavage domains. These modalities are more difficult to scale than CRISPR technologies, because new protein constructs must be designed for each gene targeted.

Therefore, for mammalian cells in particular, when tagging of endogenous loci is not feasible or practical, other approaches exist that result in endogenous or near-endogenous levels of protein. Bacterial artificial chromosomes containing the protein of interest under control of the endogenous promoter can be stably transfected into mammalian cells^62^ for protein expression. Ectopic promoters can also generate near-native levels of protein. For example, tetON systems, in which addition of tetracycline derivatives drive gene expression, are titratable, which allows researchers to control the level of ectopic expression.^63^ These ectopic genes can be introduced into cells via transient transfection, random generation of stable clones, or episomal vectors, which are maintained in the nucleus in a nonintegrated state and replicate, allowing generation of a semistable cell line.^64^ Lentivirus and adenoviruses can also be used to generate stable clones by integration into the mammalian genome; however, random integration or integration of multiple copies of the gene can lead to disturbances in cellular physiology and uncontrolled gene silencing. As a solution, ectopic genes can be targeted to “safe harbors” in the human genome,^65^ such as AAVS1,^66^ where integration of genes has been shown to minimally perturb cellular physiology or gene regulation.

Regardless of how the DNA coding for the tagged protein is introduced, some form of selection is typically required to generate cell populations containing the tagged protein. Most often this is done using antibiotic selection, where along with the desired mutations an antibiotic resistance cassette is introduced.^44,45,48,50^ Fluorescent proteins can also be added which enable cell sorting for the desired tagged proteins.^67^ Both of these methods do involve expression of exogenous proteins by the cell. Single cells can also be isolated and sequenced to cultivate clonal strains with a single mutation; this enables the addition of a tag without need for additional selection cassettes.^47^

### 2.5 Cross-Linking to Preserve Noncovalent Interactions

Depending on the mode of MS analysis chosen, it may be helpful to add a cross-linking step as part of the purification process. Cross-linking refers to covalently linking together reactive amino acids in proteins and protein complexes.^68−70^ Cross-linking can be coupled to any of the three enrichment strategies discussed above (Figure 3) and enables samples to be purified stringently while retaining weak but physiological associations. Cross-linking generally increases the number of proteins that copurify^71,72^ and is also used to generate distance constraints for cross-linking MS (see section 3.3.2.1).

A wide range of cross-linkers exist,^73^ and the choice of cross-linker will depend on downstream application. Cross-linker concentration and reaction time should be optimized to ensure that only physiological interactions are captured. Formaldehyde and glutaraldehyde are two popular small, cell-permeable cross-linkers that are useful for simply preserving interactions, with glutaraldehyde functional at low temperatures.^74^ Other cross-linkers are designed for subsequent cross-linking MS analyses, as reviewed in ref (73) and discussed below (section 3.3.2.1), many of which are also cell permeable. Some cross-linkers can be affinity purified from cell lysate via reactive handles, often based on click chemistry, for the enrichment of otherwise low-abundant cross-linked peptides from complex biological samples.^75^

Cross-linking can occur at many points throughout the purification procedure. Cell-permeable compounds can be added before cellular lysis. For example, in rapid immunoprecipitation mass spectrometry of endogenous protein (RIME), a protocol developed by Carrol and co-workers, in-cell formaldehyde cross-linking followed by immunoprecipitation and MS analysis is used to identify protein complexes.^76−78^ Intriguingly, Fabre and co-workers^79^ found that cross-linking before cellular fractionation prevented leakage of proteins between different fractions and preserved native localization of proteins after fractionation. Cross-linking can also take place in lysates,^80^ in cell powders,^74^ or after complexes are captured on affinity beads^82^

Chait and co-workers have engineered lysine-free anti-GFP nanobodies for on-bead cross-linking with lysine cross-linkers; the anti-GFP nanobodies will not be affected by cross-linking.^82^ Cross-linking can also be performed in gel after native gel separation^83^ of endogenous complexes. However, it is important to note that Zhang et al. found different cross-linking results between in-cell and in-lysate linking;^71^ therefore, for optimal preservation of biological interactions, cross-linking should take place before cell lysis.

In addition to adding exogenous cross-linkers, photo-cross-linking amino acids can be incorporated into cellular proteins and used to capture protein–protein interactions in cell following light activation.^84^ Photoleucine,^85^ photomethionine,^85^ and photolysine^86^ are incorporated into all cellular proteins, with rates ranging from 4% to 40% and have been used for MS identification of histone binding proteins. Site-specific incorporation of photoreactive amino acids into specific proteins can be achieved using unnatural amino acid incorporation^87^ or split intein technologies^88^ to specifically interrogate the interactome of one protein, such as the membrane protein IFITM3 for which traditional affinity purification methods failed to recover true interactors.^89^

## 3 MS-Based Techniques for Studying Endogenous Complexes

### 3.1 Biological MS Delivers Insights Across Multiple Levels of Complex Regulation

A range of biological MS techniques exist which differ in the details of sample preparation and data analysis but have in common the generation of gas-phase ions of proteins or protein fragments, mass separation, and detection (Figure 2). Techniques can be classified as either “bottom-up”, in which proteins are digested into peptides before gas-phase ion creation, or “top-down”, in which proteins are transferred intact into the mass spectrometer.^90^ Middle-down approaches, in which proteins are partially digested into large fragments (>7 kDa^90^) are also emerging but will not be discussed further here. In all forms of MS, specific ions can be selected for gas-phase ion fragmentation in tandem MS experiments.^91,92^ By fragmenting ions and analyzing the daughter ions, sequence and structural information about the parent ion can be determined.

We will cover a range of bottom-up approaches, including shotgun proteomics, cross-linking MS, HDX MS, chemical labeling MS, as well as top-down MS and native-MS. After providing a brief overview of each technique, highlighting its pros and cons, we will describe what information it can provide for endogenous complexes. By combining different sample preparation protocols described above with these varied MS modalities, many different types of information can be extracted (Figure 1).

For a given complex, it is important to determine both its *composition*, namely the distinct subunits that form the complex, as well as the *stoichiometry* of those proteins, namely how many copies of each subunit is present. The subunit interaction network, describing which proteins specifically interact, should be defined, and subunits classified as core or peripheral. Under the core-attachment model, proteins form core complexes, which have a stable, permanent relationship but are often temporally regulated through the attachment of peripheral proteins.^93−95^ Members of the core complex typically share functional annotations, protein localization, and even specific deletion or mutational phenotypes, while attachment members do not necessarily share these properties. The interaction of attachment members with the core complex is highly controlled. Bottom-up proteomics, cross-linking MS, and native-MS can inform on complex composition and stoichiometry for both core and attachment complexes, although the former are easier to preserve through an MS work-flow.

A given genetic sequence can produce multiple proteoforms, a term which refers to a defined sequence of amino acids with localized modifications.^96^ Different proteoforms arise due to alternate initiation sites, splicing events, and post-translational modifications (PTMs). PTMs are chemical modifications that occur following protein biosynthesis.^97^ As many as 300 PTMs are known to occur physiologically^97^ and can range from addition of small acetyl groups to conjugation of ubiquitin and other signaling proteins. PTMs are a key way that the cell temporally regulates the activity of different proteins and protein complexes, with evidence suggesting that proteins at the center of interaction networks are more likely to be post-translationally modified.^98^ Analysis of PTMs as a function of cellular state is an important component of understanding protein complex biology. PTMs clearly manifest in the MS spectrum as shifts in protein and peptide mass, and both bottom-up and top-down MS can inform on the PTMs of specific complexes and on large-scale proteome wide screens of PTMs.

MS-based methods, in particular cross-linking MS and ion mobility (IM)-MS, can also determine the topologies of protein complexes, which is especially relevant for endogenous, heterogeneous complexes available in limited quantities that prohibit high-resolution structure determination. For large complexes, if high resolution structures of individual subunits are available, they can be combined with MS-based topologies to create a 3D map of the entire complex. MS-based information can be converted into restraints for a particular protein or protein complex and then used to determine structures and structural ensembles, often in combination with computational modeling tools (see section 3.5).

In addition to determining static topologies and structures, complexes undergo conformational fluctuations as they move between different functional states. Footprinting MS techniques can inform on these conformational fluctuations and the ensemble of different structures present in solution.

Lastly, MS methodologies can be used to study the binding of cofactors and ligands to complexes. It is especially important to identify which small molecules are bound to the complex in its endogenous state and to unravel how these small molecules regulate complex activity. Identifying small-molecule binding is primarily accomplished via native-MS, but information can also be extracted from specialized bottom-up experiments.

### 3.2 Sample Separation and Fractionation

The power of mass spectrometry as an analytical tool stems from its ability to distinguish between components with different *m*/*z* ratios, enabling the detection of the full distribution of coexisting states of a given peptide or protein complex. However, like many analytical techniques, MS has a limited dynamic range,^99,100^ which causes intense ions to suppress the ionization, and consequently signal, of weaker, less abundant ions. The signal of protein and peptide ions can also be suppressed by the presence of salts and other matrix components.^101^ Spectral complexity is also a challenge and congested spectra containing overlapping peaks are difficult to analyze and assign.

Extensive sample fractionation is used to combat these challenges. By separating samples into different fractions, broader coverage can be achieved. Chromatographic separations can also reduce ion suppression by matrix sample components by enabling effective buffer exchange of the desired analytes. As mentioned above, different types of chromatographic separations are an important component of isolating endogenous complexes; however, here we focus on separation methods that typically constitute part of the MS analytical workflow, acknowledging that they overlap to some extent.

#### 3.2.1 Electrophoresis-Based Methods

MS proteomic analysis of protein mixtures was first coupled to polyacrylamide gel electrophoresis, or PAGE, as an offline chromatographic separation tool. In these experiments, protein spots were excised from gels, either Coomassie or silver-stained, and subjected to in-gel digestion,^102^ followed by LC/MS analysis of the digested peptides.^103^ Most typically, 2D PAGE was used,^104,105^ in which proteins are first separated according to isoelectric point and then by molecular weight. However, its practicality is limited by the fact that it requires extensive sample handling, is time-consuming, and cannot be coupled directly to the mass spectrometer. Moreover, 2D PAGE has limited ability to identify medium-to-low abundant proteins.^106^ 2D PAGE also has limited loading power and a resolving power limited by the combination of isoelectric focusing and molecular weight. Therefore, it has been primarily supplanted by liquid chromatography methods for MS analyses, although some groups continue to advance methods for increasing the separation efficiency of 2D PAGE.^107^

A related electrophoretic method is the GELFrEE method, or gel-eluted liquid fraction entrapment electrophoresis, which also separates proteins based on molecular weight.^108,109^ In GELFrEE, proteins elute off the end of a polyacrylamide gel column and are trapped in a collection chamber in multiple fractions. While GelFREE is not as robust as other fractionation methods,^110^ it has the advantage that it can be performed in denatured or native mode,^24^ permitting size-based separation of native proteins for MS applications.

#### 3.2.2 Liquid Chromatography

Liquid chromatography, most commonly high-pressure liquid chromatography (HPLC), is one of the most popular methods for protein and peptide separation prior to MS/MS analysis. It is especially useful because, in combination with electrospray (ESI) methods of ionization, it can be directly coupled to the inlet of the mass spectrometer. Thus, samples can be simultaneously separated and exchanged into MS compatible buffers. Programs can be controlled by software, leading to high reproducibility and easy optimization.

The most popular HPLC method to couple to an MS system is reversed-phase HPLC(RP-HPLC).^111^ RP-HPLC separates proteins and peptides based on hydrophobicity, using a hydrophobic stationary phase and eluting with organic solvents. These organic solvents are MS-compatible, making RP-HPLC an excellent tool for MS analysis. RP-HPLC also offers good resolution, easy tuning of elution conditions, and is particularly suited to peptide analysis because peptides are typically recovered well from RP columns. However, RP-HPLC requires denaturing and is not applicable to native-MS applications; moreover, recovery of intact proteins is variable.

For complex proteomic samples, 2D LC/LC/MS experiments increase resolution with multiple dimensions. For example, MudPIT, introduced by Yates and co-workers in 2001, combines strong cation exchange chromatography with RP-HPLC to separate and detect a wider range of peptides.^112^ Generally, ion exchange columns can also be used for fractionation but suffer in terms of coupling to the mass spectrometry because the salt used for elution will be injected into the MS system, causing severe signal suppression. Directly coupling ion exchange columns to the mass spectrometer requires the use of MS compatible volatile buffers such as ammonium acetate for ion exchange. Therefore, for analysis of peptides, RP-HPLC is almost always used as the final step before the mass spectrometer.

LC-MS is emerging as a technique for buffer exchange^113^ and even purification^114^ of intact proteins and complexes for native-MS. In these experiments, a small size-exchange column buffer exchanges proteins into MS-compatible buffers directly prior to analysis, and tandem chromatography methods can be used to combine affinity columns with buffer exchange. Other advances in liquid chromatography for MS analysis, as reviewed in ref (115), include the development of smaller columns which enable faster separation and introduction of new resins.

#### 3.2.3 Capillary Zone Electrophoresis

Capillary zone electrophoresis (CZE) separates ions in solution based on their electrophoretic mobility under applied voltage.^116^ The electrophoretic mobility is dependent on charge, shape, and size of proteins or peptides. CZE provides orthogonal, complementary data to liquid chromatography. Advantages of CZE include low sample requirement and low carry-over between separation experiments. It also provides relatively fast separation, which can be an advantage or a disadvantage, because analytes can elute too quickly to be separately fragmented in tandem MS experiments.

CZE has been applied to proteomics workflows in bottom-up mode, detecting patterns of PTMs^117^ in endogenous proteins and to rapidly separate peptides in complex proteome digests.^118−120^ However, a back-to-back comparison of CZE and RPLC indicates that RPLC may identify more peptides and post-translational modifications,^121^ and CZE has limited loading capacity. Both methods can be combined for increased separation capacity.^121^

CZE can also be used to analyze proteins in top-down mode, demonstrating separation of antibody variants differing by single deamidation events.^122^ Importantly, CZE is compatible with separation under native conditions^123^ in which proteins retain their tertiary and quaternary structure. Recent work by Kelleher and co-workers applied native CZE to analysis of endogenous nucleosomes,^124^ using tandem MS to characterize differences in proteoforms between endogenous nucleosomes in different cell lines.

#### 3.2.4 Ion Mobility

Ion mobility (IM) separates protein and peptide ions within the mass spectrometer after gas phase ionization. In IM experiments, ions traverse a gas-filled tube and are separated according to their collision cross section. The collision cross section is a property of the ion-gas pair and has dependence on the charge, size, and shape of the ion.^125^ An extended ion will experience more collisions with the gas and thus travel more slowly than a ion with the same mass but a more compact structure.^126−128^ There are multiple IM MS devices, such as drift tube, traveling wave, differential mobility, transversal modulation overtone, field asymmetric, and trapped IM-MS, each with its own advantages and disadvantages, and we refer the readers to detailed reviews on this topic.^129,130^

For analysis of peptides in bottom-up proteomics experiments, including IM greatly increases the detection capacity of the experiment,^131^ enhancing selectivity, resolution, and dynamic range by spreading peptides out over an addition dimension.^132,133^ Addition of IM to proteomic workflows advances the ability of proteomics to study increasingly smaller quantities of protein^134^ toward the goal of single-cell proteomics.

IM is also a powerful tool for native MS analysis of proteins. Particularly, it is used not only to separate sample components but also as a structural biology tool. Extraction of collision cross sections from IM data constitute a restraint that can be used to generate information the 3D shape and conformation of protein complexes^135^ (see below sections 3.4 and 3.5).

### 3.3 Bottom-Up Approaches

#### 3.3.1 Proteomics

##### 3.3.1.1 Overview of Bottom-Up Protein Identification

The most popular form of biological MS is bottom-up proteomics, described in detail in refs (6, 136, and 137). For bottom-up proteomics experiments, proteins are digested into peptides by proteases, trypsin being by far the most popular. Trypsin’s popularity stems from the fact that it is an extremely efficient protease that cleaves after basic arginine or lysine residues, producing peptides that are efficiently ionized and fragmented in tandem MS experiments. However, because of its cleavage specificity, trypsin has a bias toward hydrophilic portions of the protein. Higher sequence coverage and complementary data sets,^138^ particularly in terms of PTM identification, can be accomplished by digestion with complementary enzymes, including chymotrypsin, LysC, LysN, AspN, GluC, and ArgC, as reviewed in ref (139). Enzymes can also be sequentially applied to improve protein identification.^140^

As discussed above, peptides are then typically separated on RP-HPLC columns and injected into the mass spectrometer. Tandem-MS techniques^91,92^ are then applied for sequence analysis via gas-phase fragmentation patterns. Different strategies for choosing which peptides to analyze via tandem-MS strategies are available, as even with the best liquid chromatography separation strategies, a large number of peptides elute simultaneously. In targeted proteomics, or selected reaction monitoring experiments, specific ions corresponding to proteins of interest are chosen a priori for fragmentation and quantification.^141^ These experiments are fast, reproducible, and extremely sensitive to the protein of interest but cannot be used to discover new proteins. For analysis of complex mixtures, researchers rely on shotgun proteomics methods which attempt to sequence all proteins in complex peptide mixtures. In data-dependent acquisition (DDA), the mass spectrometer selects peptides meeting a certain threshold in the first MS scan and then selects them for fragmentation. DDA is the most widely used method for shotgun proteomic analysis and it aids peptide identification because the precursor mass of the ion is known. However, it is estimated that an extremely large percentage of peptides are missed,^142^ prompting the development of data independent acquisition (DIA) strategies. In these methods, first proposed by Yates and co-workers,^143^ specific ranges of the MS spectrum are selected sequentially for tandem MS analysis automatically. DIA holds promise for better proteome coverage^144^ but the data produced is significantly more complicated. Different variations of DIA are a promising area of research to advance shotgun protoeomics.^145,146^

Protein amounts can also be quantified via quantitative proteomic methods. Quantification methods generally fall into label-free methods, which include peak intensity based methods and spectral counting^147^ or through isotope labeling. Peak intensity based methods, such as iBAQ,^148^ rely on integrating peak intensities, which were shown to correlate well with protein concentration.^149^ In spectral counting,^147^ the number of peptide spectra assigned to a protein of interest is used as a proxy for relative protein abundance. Generally, peak intensity based methods are considered to be more accurate than spectral counting, but both have limitations because they rely on accurate identification of peptides across multiple spectra. The gold standard for relative quantification is the isotope labeling methods. These methods rely on the fact that peptides with the same sequence but different isotope enrichment will coelute but be separated in a single spectrum for quantification. Labeling methods include stable isotope labeling in cell culture (SILAC), in which proteins are grown in isotope enriched media,^150^ or the addition of mass tags to samples postisolation.^151,152^

After the MS data is generated, significant software analysis is required to identify and quantify proteins in the MS spectra; choice of data processing strategy is an integral component of experimental design. The tandem-MS experiments must be analyzed to determine the peptides present, the proteins they belong to, and to quantify their percentages. Reference (153) describes some of the different strategies used for analyzing shotgun proteomic data. Typically, MS/MS spectra are collected and compared to databases involving all spectra corresponding to a digest of the parent proteome, either theoretical or experimentally generated. Software with this capability include MASCOT,^154^ MaxQuant,^155^ Progenesis, and PEAKS.^156^ However, de novo sequencing, or forward prediction of peptide sequence based on tandem-MS data, is also an option, with other softwares incorporating this capability.^157^ When analyzing targeted proteomic data, Skyline is the most widely used.^158^ The ProteoWizard toolkit provides an open source platform for converting data from different mass spectrometer formats and cross-platform analysis.^159^ Several groups have compared the different available softwares and shown that the software and data processing pipeline chosen has a significant impact on the results of the LC-MS/MS analysis,^160,161^ both finding that with suitable data processing, MaxQuant is the most robust. These researchers also demonstrated, as expected, that for quantification, targeted MS has a higher dynamic range and limit of linearity because specific peptides are targeted and chosen.^161^

For proteomics-based methods, the absolute sensitivity threshold is quite low; a modern mass spectrometer is able to detect as little as 1 × 10^–18^ mol of peptide.^162^ Other sources of error throughout the workflow lead to proteins being missed or misidentified. In the sample preparation step, poor cellular lysis, poor solubilization of proteins, and contamination from abundant proteins, keratin, or dust are all significant sources of error. As mentioned above, another challenge is the dynamic range of the proteome; as discussed in ref (100), the dynamic range of MS is only 4 orders of magnitude while that of the proteins in the cell is 7 orders of magnitude at least. Therefore, many low abundant proteins, which may constitute real interactors, are missed in analyses of protein interactomics, and enhancing proteome coverage is a major challenge for bottom-up proteomics. The limited ability to select and fragment peptides can also cause peptides to be missed, even if they are present in the spectrum, and moreover, the software can misidentify or miss peptides that are present at low signal-to-noise levels. Therefore, when analyzing a particular endogenous protein complex, users should be aware that low-abundant or transient interactions may be missed even in these extremely sensitive experiments.

One area of advance in proteomics is focused on platforms that can analyze proteins from single cells. Termed “single cell proteomics”,^163,164^ advances towards this effort include development of miniaturized automated nanodroplet processing platforms capable of bottom-up digestion of single cells,^165,166^ usage of narrow-bore LC-MS^167^ and new methods for cell lysis.^168,169^ This increased sensitivity has relevance for studying complex heterogeneity at the single cell level.

In addition to identifying and quantifying proteins, bottom-up proteomics can identify a wide range of PTMs, which manifest as shifts in peptide mass and fragmentation patterns.^170,171^ However, a number of pitfalls exist in the use of bottom-up proteomics to identify PTMs, reviewed in ref (172). Information on the coexistence of different modifications in a single polypeptide is lost because a mixture of peptides is generated and analyzed together. Similarly, it is difficult to determine which proteoforms coexist in a complex together because the bottom-up results only report on the presence or absence of the PTM. Therefore, while post-translationally modified proteins are often detected in bottom-up proteomic experiments of protein complexes, including *in cell* cross-linking experiments,^173^ it is difficult to functionally characterize how PTMs regulate complex formation from bottom-up data alone.

##### 3.3.1.2 Bottom-Up Proteomics Illuminates Complex Composition

**Figure 4:**
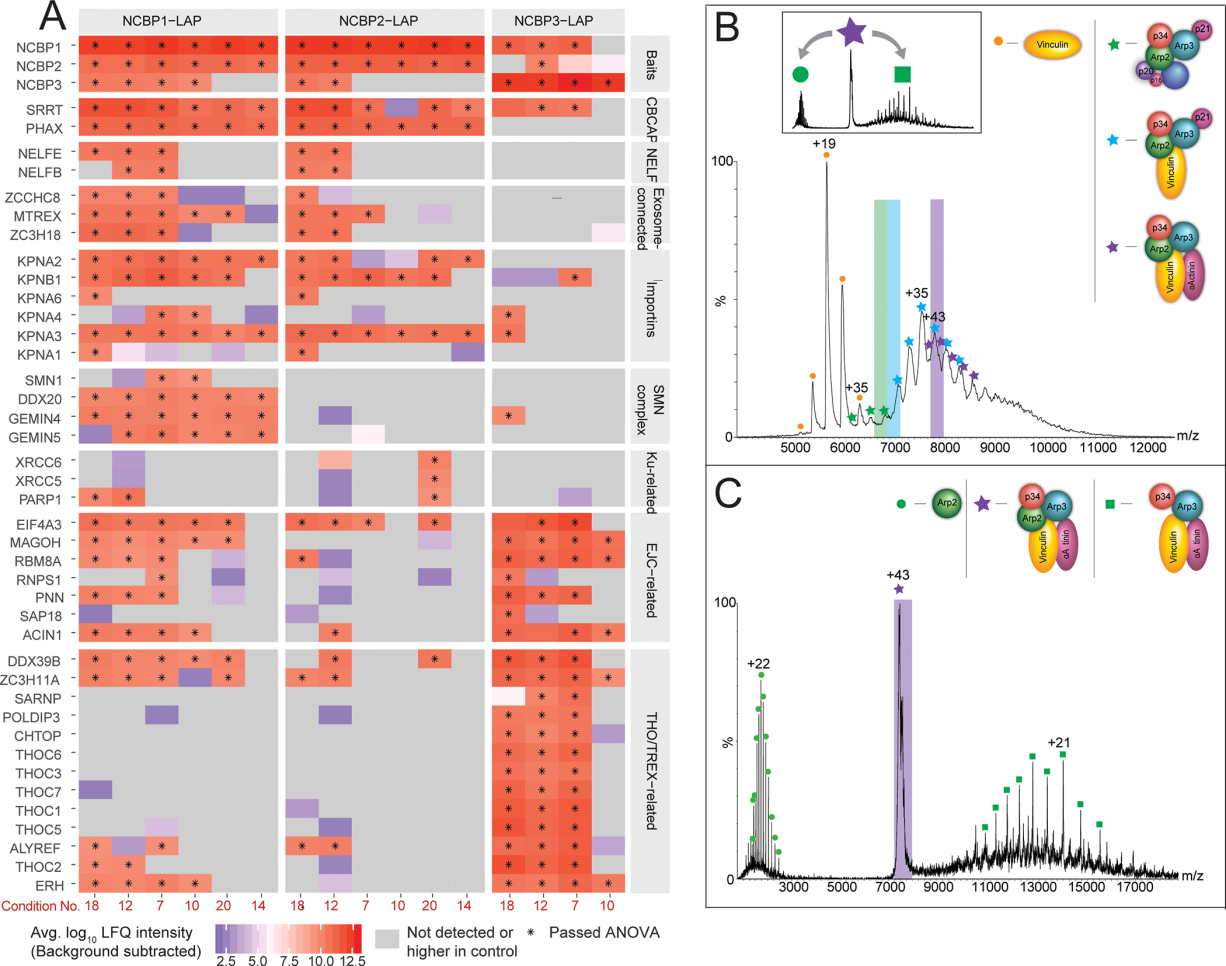
Identifying complex modularity via bottom-up proteomics vs native MS. (A) In bottom-up proteomics experiments, complexes are revealed by identifying copurified proteins. Here, three different tagged proteins were used as “bait” (top row), namely NCPB1, NCBP2, and NCBP3, which are different nuclear cap binding proteins that bind to nascent RNA. The data can be visualized as a heatmap of control-subtracted label free quantification (LFQ) intensity for each “prey” protein identified by MS. This data demonstrates that NCPB3 differs from NCBP1 and NCBP2 in selectivity, coprecipitating with members of the THO/TREX complexes. These interactions were later confirmed as supporting a biological role for NCBP3 in mRNA expression that differs from NCBP1 and NCBP2.^352^ (A) Figure reproduced and adapted with permission from ref (353). Copyright 2020 Oxford University Press. (B) Shown is a native mass spectrum of multiple coexisting complexes purified from chicken muscle. While the main charge series corresponds to monomeric vinculin, additional charge states corresponding to three protein complexes are also seen. The masses of these complexes indicate the presence of intact Arp2/3, a vinculin-associated Arp2/3 complex, and a vinculin-α actinin-associated Arp2/3 complex. (C) Tandem-MS is used to validate the complex composition; shown is the MS/MS spectrum of the charge state selected in purple, with circles denoting released subunits and squares corresponding to the remaining stripped complex. Tandem MS reveals that Arp2 is released from a complex composed of vinculin, α-actinin, and the Arp2/3 complex. (B,C) Reproduced and adapted with permissions from ref (18). Copyright 2014 Springer Nature.

In terms of characterizing endogenous protein complexes, bottom-up proteomics is typically used to provide information on the complex composition when investigating a particular complex or configurational differences that occur under different cellular states by comparative analysis of multiple conditions (Figure 4A). In this experimental format, protein complexes are predicted from observation of which peptides, and therefore proteins, are detected as copurifying. The experimental pipeline typically involves a method of copurifying proteins under native conditions, followed by bottom-up MS analysis of the purified fractions and computational filtering to predict complexes on the basis of which proteins copurify.

One method of generating complexes for copurification identification is intensive biochemical fractionation. This has been used to fractionate lysates from a range of cell types,^21,23,174−176^ with cofractionating proteins identified by MS and endogenous complexes predicted by observing which proteins cofractionate. Importantly, multiple studies corroborate that many proteins elute at a MW larger than that predicted by their sequence, implying preservation of native complexes throughout native extraction methods.^23,176^ In a recent example, Kastritiis et al. used SEC to characterize endogenous protein supercomplexes, a term that refers to megadalton-sized macromolecular assemblies composed of multiple functionally related protein complexes, from *Chaetomium thermophilum*.^176^

More popularly, co-IP, followed by MS identification, is used to identify protein–protein interactions and thereby infer protein complex composition (see ref (177) for a review). Several high-throughput, broad studies combining IP and MS have been conducted which provide excellent starting resources for understanding cellular interactions and complexes. These include endogenous TAP tag based studies in yeast,^95,178^ which identified 491^95^ and 547^178^ heteromeric complexes. Endogenous human complexes were IP’ed from HeLa cells using over a thousand primary antibodies^179^ and previously unknown complexes identified, including a complex suggested to be involved in transcriptional coregulation. Human affinity-tagged lines were also created, with Hein et al.^180^ using BAC transgenes while the Gygi lab took advantage of the human ORFeome project^181^ to create lentiviral lines coding for a wide range of human proteins in HEK and HELa cells.^182,183^ Both of these studies yielded rich data sets that map a wide range of interactions connecting thousands of endogenous proteins.

To determine complex stoichiometries via copurification and bottom-up proteomic experiments, subunit stoichiometry must be inferred from relative abundance of peptides corresponding to different proteins. Label-free quantification of peptides can determine relative abundances of different proteins and was used to determine the stoichiometry of NPC complexes,^184^ chromatin-associated complexes,^185^ and SET/MLL complexes,^186^ to give some examples. Information about total cellular protein abundance can also be incorporated to extract subunit stoichiometries^180^ because the amount of protein that copurifies with a particular bait will depend on the cellular abundance of the interacting components as well as on the stoichiometry of the individual complex.

The computational approach used to predict protein complexes is a key component of the experimental workflow. As reviewed in refs (187−189), a variety of strategies exist to generate complexes from protein–protein interaction lists. For example, one algorithm, SAINT-MS,^190^ uses a Bayesian model to estimate the probability of a given interaction being true or false, incorporating information from negative controls and spectral intensities, while another, COMPASS, incorporates information about spectral counts and reproducibility to score interactions.^191^ However, it should be mentioned that poor reproducibility was found between different algorithms applied to the same data set.^192^ Including orthogonal pieces of information, such as gene ontology annotations and gene expression data, with MS-derived interaction data can increase reliability and help remove false positives.

Users should be aware that affinity copurification methods to identify protein complexes can suffer from lack of reproducibility, false positives, and false negatives. Gavin^95^ et al. found that duplicate IP-MS measurements from budding yeast had only 69% replicability, although others found that the same protocol conducted by two laboratories had 81% reproducibility.^193^ In a meta-analysis of three data sets,^21,180,183^ Drew and co-workers observed limited overlap between different high-throughput studies^194^ with many false positives. False positives, or contaminants, generally result from nonspecific binding to either the bait protein or the affinity resin used. Specific isotope enrichment strategies, such as I-DIRT, can distinguish contaminants,^195^ and the CRAPome is a repository of common contaminants for different affinity purification strategies^196^ that should be checked against identified complex components to identify contaminants. False negatives in affinity-purification MS measurements generally result from complex dissociation during purification, from poor tryptic digestion of peptide interacting partners, or from data undersampling in the mass spectrometer.

Besides for high-throughput experiments, affinity-purification proteomic experiments have broad applicability to studying specific proteins and their formed complexes under conditions as close to endogenous as possible. In an early example, Yates and co-workers analyzed proteins present in the ribosome by ribosome purification and bottom-up proteomic analysis of all present components.^197^ Examples of proteins studied span the gamut of cellular proteins, from identifying shared targets of an E3 ligase substrate receptor^198^ to the RNA exosome^199^ and LINE-1 transposon elements.^200^Figure 4A describes a recent study examining interactions of different RNA cap binding proteins that revealed a unique role for one protein in regulating mRNA expression.

An emerging bottom-up proteomics-based method to identify endogenous complexes via MS is thermal proteome profiling, or TPP. In this experiment, whole cells lysates are subjected to a temperature gradient, and then protein abundance as a function of temperature is measured via isotope quantified bottom-up proteomic methods to construct a melting curve for each protein. Although first described as a method for identifying drug-induced protein stabilization,^201^ correlation of melting profiles for different proteins, termed thermal protein coaggregation (TPCA),^202^ can identify associations based on the assumption that bound proteins have correlated melting curves. In the initial report, 30% of CORUM qualified complexes exhibited correlated TPCA curves.^202^ TPCA is less useful for de novo complex prediction and also suffers in the analysis of low-stoichiometry complexes, for whom melting curves may be dominated by the noncomplexed protein. Nevertheless, TPCA analysis is particularly powerful for analysis of differential protein complex association between states such as throughout the cell cycle^203^ or at differing time-points following virus infection.^204^

#### 3.3.2 Protein Footprinting

The sample separation and analysis strategies outlined above represent a powerful tool for detecting and analyzing peptides originating from protein complexes. A wide range of strategies, broadly dubbed “protein footprinting”,^205,206^ use chemical or enzymatic sample treatment before MS analysis to encode information about the protein complex’s structure, topology, and interactions in the MS spectrum. The vast majority of the time bottom-up proteomic methods are used to detect these chemical modifications, but top-down methods have be used occasionally.^207,208^

##### 3.3.2.1 Cross-linking MS

###### 3.3.2.1.1 Overview of Cross-Linking MS

In cross-linking (XL) MS, reviewed in refs (68 and 209), samples in their native state are treated with bifunctional cross-linkers that react with reactive amino groups, acidic side chains, or cysteine groups. These cross-linkers will covalently link together amino acids that are proximal in the three-dimensional structure of the protein or protein complex. After digestion, peptides generated will be composed of two smaller peptides linked by the cross-linker. LC-MS/MS detection and quantification of cross-linked peptides yields information about intra- and intermolecular linkages between residues. Chavez and co-workers estimate the sensitivity of cross-linking MS to be 1–10 × 10^–12^ mol for purified proteins and 5–10 mg of sample for in situ cross-linking,^210^ making it an attractive option for endogenous complexes.

The choice of cross-linker is crucial to the XL-MS experiment; as reviewed in ref (73), a wide range of reagents exist with qualities such as distinctive tandem-MS fragmentation patterns^211−213^ to enable clear identification of the cross-linked peptide among the background peptides. The next step, and the main challenge in XL-MS experiments, is to accurately analyze the cross-linking reaction mixture via MS and identify and quantify the peptides. Productive cross-linked products consist of two connected peptides, resulting in a quadratic expansion of the search space for the typical bottom-up proteomic algorithm. However, off-target peptides consisting of a single peptide attached to a cross-linker can also be produced. Moreover, cross-linked peptides may not fragment ideally in the MS/MS process, leading to poor signal-to-noise for one of the constituent peptides. Different softwares exist for the analysis of cross-linking MS data; XlinX^80^ and Merox^13^ are focused on MS-cleavable linkers, while Xquest,^214^ Kojak,^215^ StavroX,^216^ and pLink^217^ can be used for noncleavable linkers. A recent comparison of different softwares using a benchmarked library of peptides demonstrated that false discovery rates vary greatly depending on the software used, with FDR rates ranging from 2.4 to 32%.^218^ Reference (219) discusses future initiatives to make XL-MS more reliable and reproducible.

Once cross-linked peptides have been accurately detected and quantified, they can be converted to distance restraints based on assumptions about the maximal distance between cross-linked residues and used to determine complex topology. One challenge is generating sufficient cross-links to properly orient subunits within the complex; Chait and co-workers estimate that at least 4–5 cross-links are needed to determine dimer orientation.^220^ Combining orthogonal cross-linkers^221,222^ can generate enough cross-links to reliably determine the architectures of affinity purified complexes. As discussed below (section 3.5), cross-linking MS is also commonly combined with computational modeling^223^ to derive full structures and architectures of endogenous complexes. For example, the MXNL web server was developed for model validation using restraints from cross-linking MS.^224^

###### 3.3.2.1.2 Application to Endogenous Complex Analysis

Cross-linking has illuminated endogenous protein complex composition by performing the cross-linking reaction before affinity purification to prevent complex dissociation and enrich for transient interactions. This has successfully identified interactors of M-Ras,^225^ the proteasome,^79,226^ and estrogen receptors,^76,227^ among numerous others. Cross-linking can also be used to map protein–protein interactions without affinity purification. If cross-linkers are applied to cells and cross-linked peptides subsequently identified, proteins identified as cross-linked are presumed to interact.^80^ However, this approach typically targets the most abundant proteins in the sample;^80,228^ in one example, proteins identified as cross-linked in synaptosomes were three times more abundant than the average synaptic protein.^228^

The main power of XL-MS is its ability to illuminate endogenous protein complex topology and architecture. This approach has been used to determine the architecture of diverse endogenous complexes ranging from the coatamer module of the nuclear pore complex (NPC),^220^ chromatin–protein complexes,^229^ variants of the 26S proteasome,^222,230,231^ TAP- purified PolII-TFIIF complexes,^232^ ATP-synthase,^233^ and substrate-bound TriC,^234^ among others.

**Figure 5:**
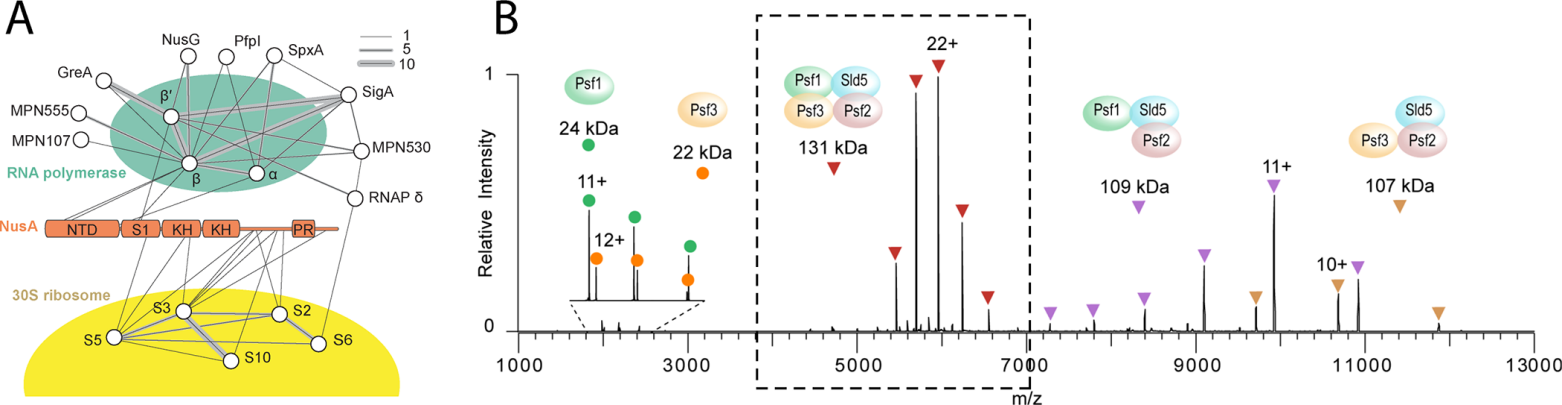
Native-MS and cross-linking MS can identify complex topology. (A) Cross-linking MS identifies the architecture of complexes via cross-links detected between subunits. Here, cross-links were generated in situ for the *Mycoplasma pneumoniae* expressome, a supercomplex composed of RNA polymerase and the ribosome linked via NusA. Cross-links oriented the C-terminal domain of NusA to RNA polymerase and the N-terminal domain to the mRNA entry site of the ribosome. A hybrid structure was later constructed that combined data from cryoEM and cross-linking MS. Reproduced and adapted with permission from ref (239). Copyright 2020 The American Association for the Advancement of Science. (B) Endogenous affinity-isolated GINS complex, a 131 kDa heterotetramer (box), was subjected to HCD activation, causing it to dissociate into subcomplexes, which appear at lower and higher *m*/*z*. By identifying the subcomplexes generated, a subunit connectivity map could be generated which was consistent with the known structures of homologous human GINS complexes. Reproduced and adapted with permission from ref (312). Copyright 2016 American Chemical Society.

One strength of XL-MS for determining complex architecture is that cell permeable cross-linkers can be added to intact cells and tissues, yielding inter- and intramolecular cross-links that reflect topologies found in vivo.^235^ Workflows for XL-MS in cells and tissues are described in refs (210 and 236). This approach has been used to determine the topology of respirasome supercomplexes directly from mitochondria,^237^ sarcomere protein complexes directly from heart tissue,^238^ and the expressome composed of RNA polymerase and the ribosome linked by NusA and NusG^239^ (Figure 5A). Rappsilber and co-workers recently demonstrated that it is possible to generate de novo cross-linked structures of bovine serum albumin in plasma.^240^ Blankenship and co-workers^241^ applied XL-MS to intact cyanobacteria, using affinity purification of photosystem II to generate a structure of the megacomplex composed of photosystem I, photosystem II, and the phycobilisome.

**Figure 6:**
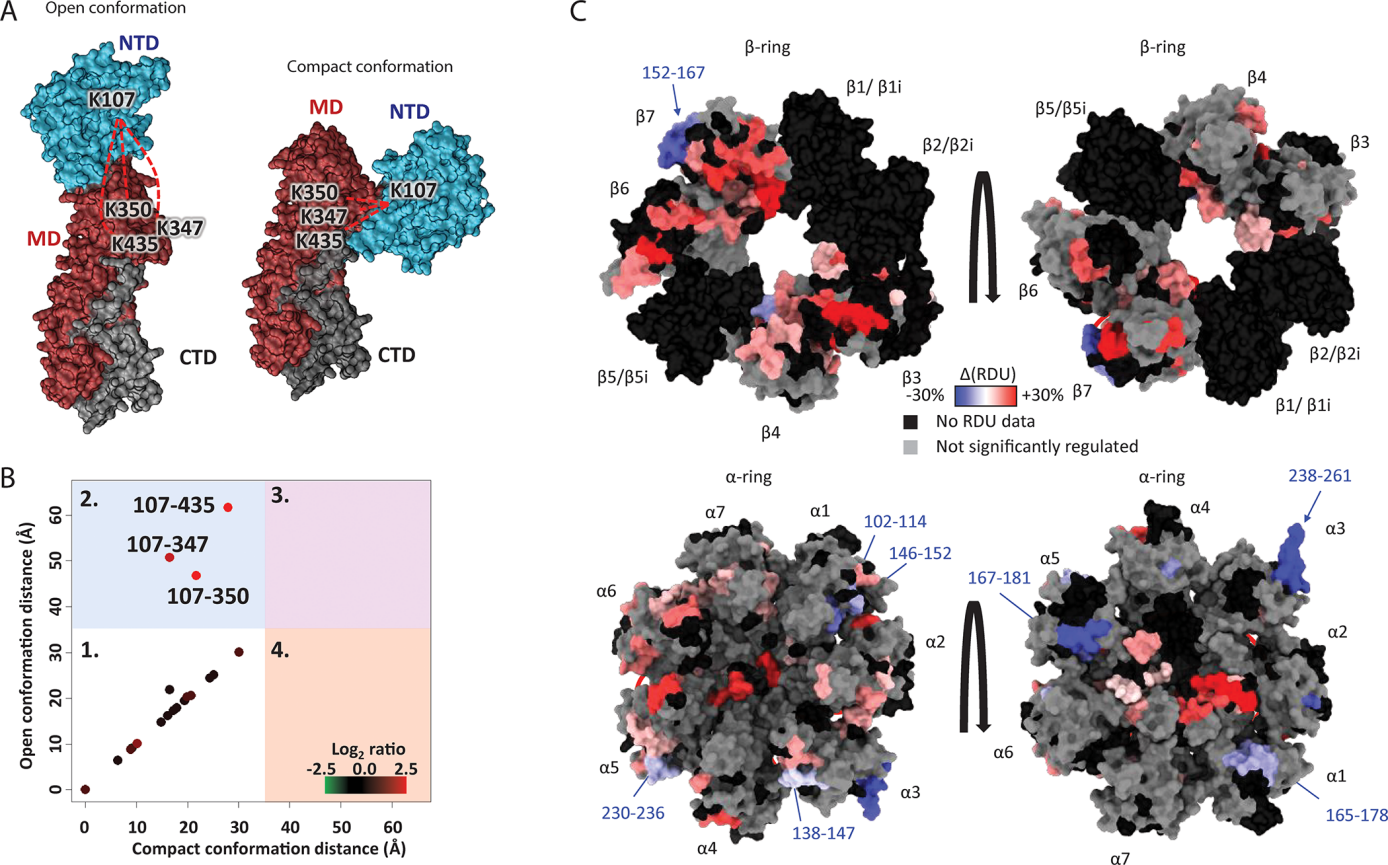
MS illuminates structural dynamics of endogenous complexes. (A,B) Cross-linking MS reveals conformational dynamics of HSP90 in vivo. HeLa cells were cross-linked, and cross-linked peptides corresponding to HSP90 were quantified with and without 17-AAG treatment. (A) Two different conformations of HSP90, with identified cross-links between the N-terminal domain (NTD) and middle domain (MD) shown in red dashed lines. (B) Plot comparing Euclidean Cα–Cα distances calculated for different HS90B experimental cross-links mapped onto the two structures: quadrant 1 is consistent with both models and quadrant 2 with compact model only. The color of the cross-links represents the log_2_ of the ratio between the two treatment conditions. This data demonstrates that the compact conformation increases in abundance after 17-AAG treatment. Reproduced with permission from ref (243). Copyright 2016 Cell Chemical Biology. (C) HDX-MS shows differences in structural dynamics between the standard 20S proteasome and the immunoproteasome. Shown is the difference in relative deuterium uptake for specific regions of the α and β rings of the immune-20S proteasome vs the standard 20S proteasome, demonstrating that some regions are more dynamic in the immunoproteasome (red) and some are more dynamic in the standard 20S proteasome (blue). The authors explained these differences as relating to differences in activity between these two proteoforms of the 20S proteasome. Reproduced with permission from ref (256). Copyright 2020 Springer Nature.

Because cross-links report on a given distance cutoff between two residues, cross-links can be a source of information on the dynamics and conformational fluctuations in a complex. Cross-links can be compared between different samples, for example, following a biological treatment. Schmidt and co-workers noted that upon dephosphorylation of the F-type ATPase, intersubunit cross-links in the head, stators, and stalk portions changed in abundance, likely reflective of phosphorylation dependent nucleotide binding.^242^ Chavez and co-workers used a quantitative XL approach in live cells to monitor changes in conformations of HSP90 upon treatment with 17-AAG^243^ (Figure 6A). Cross-links can also be analyzed to extract information about pools of a protein complex population in a single sample. The coexistence of cross-links that satisfy mutually exclusive conformations can reflect intrinsic heterogeneity and flexibility of the complex. Sinz and co-workers recently used XL-MS to examine the conformational plasticity of ribosomes, mapping a wide range of cross-links onto the model of the *E. coli* 70S ribosome and demonstrating that no single static structure satisfies all cross-links.^244^

##### 3.3.2.2 Hydrogen–Deuterium Exchange MS

###### 3.3.2.2.1 Overview of the Method

In HDX-MS, reviewed in ref (245), the “footprint” is produced by exchange of protons in a protein sample with deuterons. Protonated protein samples are incubated in a deuterium-based buffer for variable periods of time to allow exchange of amide backbone protons with deuterium in the buffer. Exchange of amide protons depends on the position of the amide site (backbone or side chain) and its dynamics (see below), with rapidly fluctuating regions exchanging quickly while rigid regions can remain stable for hours.^246^ Amide exchange is then quenched by pH and/or temperature^247^ and the extent of deuterium exchange measured. Historically, HDX exchange rates were measured by a range of methods, including NMR, but the coupling of HDX with MS readout, which easily distinguishes between deuterated and protonated peptides based on mass, greatly accelerated the application of this technique.^248^

The intrinsic exchange rate for an amide backbone is on the order of milliseconds. The rate and extent of deuterium exchange observed for a particular peptide in a folded protein, specifically reflects the local rate of hydrogen bonding for the amides in question.^249^ Generally, it can be assumed that this exchange rate is faster for amides that are solvent exposed and not involved in hydrogen bonds. If a particular region is undergoing dynamic fluctuations, local hydrogen bonds will be disrupted and potentially engage in exchange. Therefore, quantification of peptide deuteration as a function of time is generally assumed to report on solvent exposure and dynamics of a particular region. Moreover, HDX rates can be measured as a function of protein complex or protein state and therefore report on differences in subunit interfaces or dynamics between the states.

Measurements of HDX at the residue level are challenging, and therefore typically exchange rates are reported on the peptide level, meaning that this method does not have residue level information but rather information corresponding to the peptide level. HDX-MS is not be applied in vivo or in cell because following deuterium exchange for a given period of time, exchange must be quenched and the level of deuterium exchange preserved until peptides are analyzed. This prohibits sample purification after labeling and means that HDX-MS is only applicable to purified protein complexes. Much effort has been expended toward optimizing workflows to prevent back-exchange, as back-exchange during sample digestion and LC-MS analysis is a key drawback of the method. Recent developments in HDX-MS are reviewed in ref (250).

Effective HDX-MS requires approximately 100 × 10^–12^ mol of protein,^251^ which is a fairly high sample requirement relative to other MS methods. HDX-MS provides information on complex dynamics^252^ and is especially powerful when applied in a comparative format between two different states, such as upon binding of regulatory subunits^253^ or ligands.^254^ It has also been used to study protein folding and aggregation kinetics.^255^

###### 3.3.2.2.2 Application to Endogenous Protein Complexes Analysis

While most examples of HDX-MS are for recombinant proteins, it has tremendous potential for the dynamic analysis of endogenous protein complexes. In a recent example, the 20S proteasome purified from erythrocytes, as well as the immunoproteasomes purified from spleens, were analyzed and compared via HDX-MS,^256^ revealing differences in flexibility between the two isoforms (Figure 6B). The interaction of these complexes with the PA28 proteasome regulators were also studied. Another example using F-type ATPase, probed *E. coli* derived membrane vesicles, in which Konerman and co-workers used HDX-MS to examine mechanical stress in the motor during active and inactive states.^257^

##### 3.3.2.3 Chemical Labeling of MS

###### 3.3.2.3.1 Overview of the Method

As opposed to HDX-MS, which represents a reversible change (the swapping of a proton for a deuteron), footprinting via irreversible modification of amino acids is also used.^205^ This can be accomplished either with chemical modifiers or with radicals, most typically oxygen and hydroxyl radicals. These provide a permanent modification to the protein sequence that will not be lost during bottom-up proteomic analysis. Typically, solvent exposed amino acids will be chemically modified, again provided that the structure allows for reactive accessibility.

A wide range of nonradical chemical modifiers exist (reviewed in ref (205)), which react on the second to millisecond time scale. Many specifically target one type of amino acid, such as *n*-ethylmaleimide, which targets cysteine, or glycine ethyl ester (GEE), which targets aspartic and glutamic acids. Diethylpyrocarbonate (DEPC) labels a broad variety of nucleophilic residues,^258^ including histidine, tyrosine, threonine, serine, lysine, and cysteine, yielding approximately 30% coverage over the range of surface exposed amino acids in the average protein.

Radicals, and specifically hydroxyl radicals, are a more popular choice. They can modify 19/20 of the amino acids, and thus, similar to HDX, provide broader coverage over the sequence. Radicals are generally produced on the millisecond and microsecond time scales. A variety of methods for generating radicals exist, including Fenton chemistry and radiolysis of water; these methods are reviewed in refs (259 and 260). One broadly applied method of generating hydroxyl radicals, fast photochemical oxidation of proteins, or FPOP,^261^ reviewed in ref (262), generates radicals on even faster time scales of the nanosecond to microsecond time scale. This enables FPOP to be used to study protein folding events^263^ and other fast dynamic events. In FPOP, samples are incubated with H_2_O_2_, and laser irradiation of the sample creates OH radicals which broadly label nearby amino acids.

Similar to HDX, chemical labeling methodologies provide information on the solvent accessible surface area of the protein or protein complex and therefore report on the topology of the complex. They are typically applied comparatively between two states to examine changes that occur upon treatment, for example, during heat treatment of an antibody.^264^ Fast labeling approaches, such as FPOP, can illuminate fast dynamic events including protein folding.^263^

Drawbacks of these chemical labeling methods include the requirement to work in special buffers compatible with the chemical or radical of choice, as well as separation of peptides postlabeling. Moreover, it can be difficult to determine the exact location of an oxidative modification in the case of radical mediated footprinting, especially if multiple oxidations occur. Quantification of % of labeling based on spectral intensities is also difficult to assess accurately. However, Sharp and co-workers demonstrated that electron transfer dissociation fragmentation tandem MS can efficiently generate quantitative tandem MS product ions^265^ for analysis.

###### 3.3.2.3.2 Application to Endogenous Protein Complexes Analysis

Because of the irreversible nature of chemical labeling applied, the method can be applied in vivo to endogenous proteins, unlike HDX experiments. Zhu et al. applied hydroxyl radical footprinting to proteins in the *E. coli* outer membrane and studied the voltage gating of OmpF, observing changes in oxidation inside the porin upon changing the conditions of the cells.^266^ Jones and co-workers have advanced the use of FPOP to analyze proteins both in live cells^267,268^ and in *Caenorhabditis elegans*,^269^ demonstrating its potential for use as a structural technique in a multiorgan system. Chemical labeling was also applied to study human vitamin K epoxide reductase *in cell* using cysteine alkylation to determine the redox status of key catalytic cysteines.^270^

##### 3.3.2.4 Proximity-Induced Labeling

A related approach is proximity induced labeling, in which a protein of interest is genetically tagged with a protein that catalyzes the addition of a chemical tag, usually biotin, onto spatially proximal proteins. Popular proteins include APEX,^271,272^ an engineered version of ascorbate peroxidase, which is fast and quite promiscuous,^273^ or variants of biotin ligase, including BioID^274,275^ and TurboID.^276^ This technology provides information on protein–protein interactions, as opposed to topology and dynamics. Proteins can be enriched specifically if interactors are tagged with biotin or other affinity handles. However, these methods can struggle to identify specific interactors because they can promiscuously label all proximal proteins, although approaches have been developed to filter for specific interactions. For example, Krogan and co-workers combined APEX labeling with spatial reference proteins to filter for specific interactors, identifying interactors of GPCRs in response to agonist stimulation.^273^

##### 3.3.2.5 Limited Proteolysis MS

In limited proteolysis (LiP) mass spectrometry, proteins are briefly exposed to a promiscuous protease that cleaves exposed regions before denaturation and digestion for MS quantification.^277,278^ The promiscuous protein will produce a cleavage pattern, or footprint, reflective of the structure of the protein or complex. The peptides detected by bottom-up proteomics will reflect these structure-dependent cleavages. By comparing between two samples, the effects of an external condition can be monitored. Picotti and co-workers have advanced LiP MS for the unbiased analysis of crude biological samples^278,279^ by combining a promiscuous digestion step with a bottom-up proteomic workflow on a whole crude lysate. This approach has shed light on protein–metabolite interactions. By applying LiP to whole cell lysates^280^ fractionated in the presence and absence of metabolites, they detected metabolite-dependent changes in homomeric and heteromeric protein complexes, including the ribosome. These changes could be confirmed via SEC-MS profiles of proteins in the presence and absence of cofactors.

### 3.4 “Top-Down” Approaches

#### 3.4.1 Top-Down MS for PTM Analysis

In top-down MS, intact proteins or protein complexes are transferred directly into the gas phase.^281^ Once in the gas phase, tandem MS can be used to cleave proteins into fragments for sequence analysis. Top-down MS is uniquely suited to identification of proteoforms.^96^ This is because the mass of the intact proteoform is known, and thus modifications to individual amino acids can be cross-correlated with each other for an accurate description of the whole proteoform’s simultaneous or mutually exclusive PTMs.

However, top-down proteomics methods are much less developed than bottom-up methods and face a range of challenges described in ref (282), including poor solubility of intact proteins, challenges in adequate fractionation of intact proteins, low sensitivity due to dynamic range issues, low-throughput, and the complexity of data analysis. Thus, top-down proteomics often detects only the most-abundant proteins or those present at lower molecular weights. Additionally, there can be a lack of residue level sequence coverage due to poor fragmentation of intact proteins; efforts to increase fragmentation of intact proteins are ongoing.^283,284^

Top-down MS has been used to study PTMs to specific endogenous proteins, often in combination with affinity purification strategies. This strategy has been used to study glycan heterogeneity in plasma proteins,^285^ phosphorylation of endogenous mouse cardiac troponin I,^286^ post-translational modifications of pili proteins from *Neisseria meningitidis* upon host-cell contact,^287^ and examined how endogenously isolated K-Ras proteoforms are correlated with mutational status.^288^ Unbiased high throughput top-down proteomics workflows, pioneered by Kelleher and co-workers, have been used to study post-translational modifications in response to induction of DNA damage and treatment with chemical stressors^289^ as well as PTMs of cardiac proteins upon myocardial infarctions.^290^

**Figure 7:**
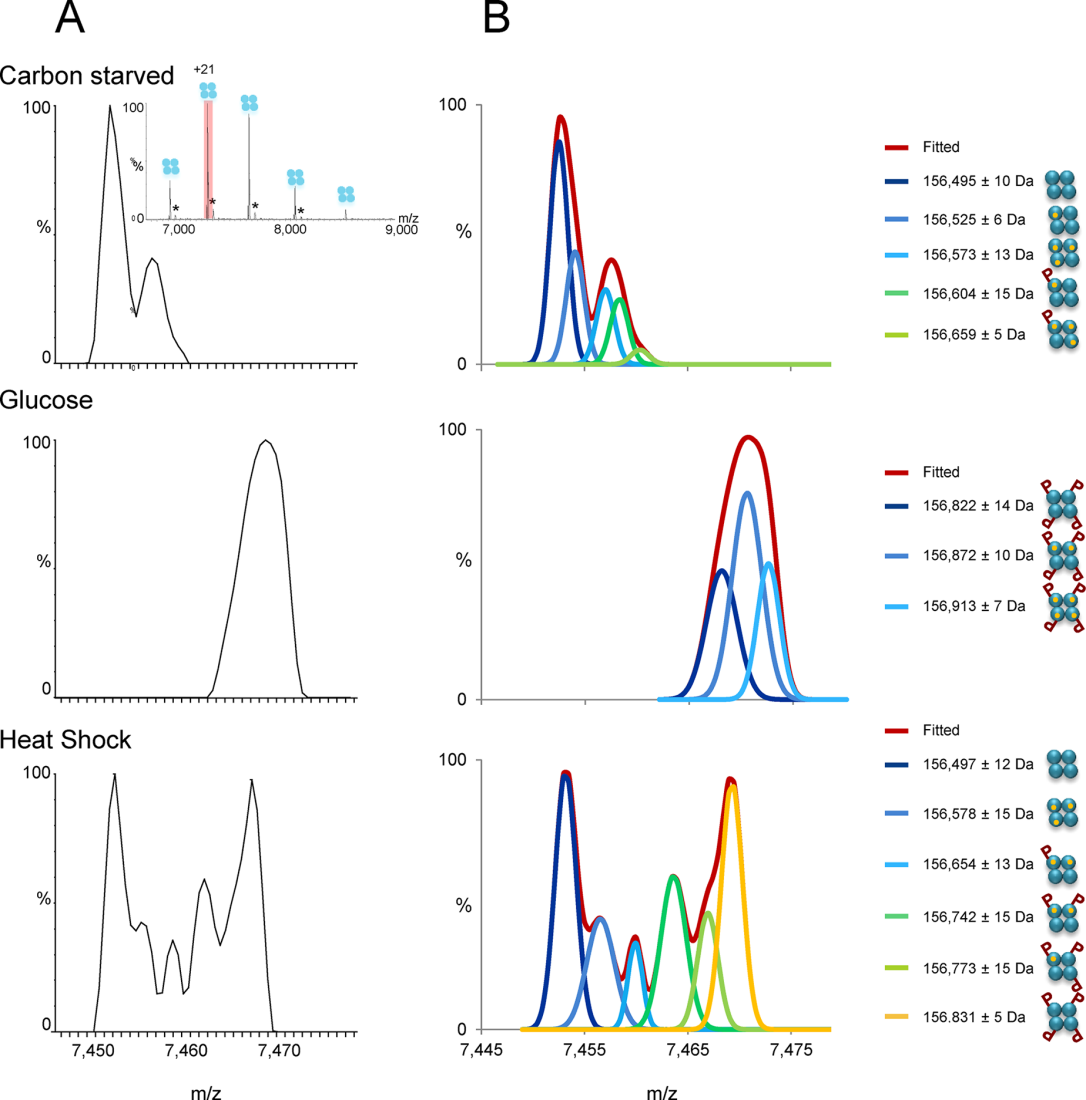
Top-down native MS identifies the proteoform composition of endogenous complexes. (A) Mass spectra of the intact intact fructose-1,6-bisphosphatase 1 (FBP1) homotetramer, purified endogenously from yeast grown under different conditions (carbon starved, glucose and heat shock). The inset shows the full charge series, and each panel displays the 21^+^ charge state for each growth condition. Spectral deconvolution (B) reveals that glucose leads to a uniformly tetra-phosphorylated, while heat shock leads to a mixture of tetramers with different numbers of phosphorylations. MS/MS analysis (not shown) revealed that each subunit is monophosphorylated in the tetramer, while MS/MS/MS fragmentation analysis localized the modifications to the ^12^Ser/^13^Thr site. Magnesium ions, known binders of FBP1, were also observed bound to the complex. Each FBP1 subunit is graphically depicted as a cyan circle. Mg^2+^ ions are indicated as small orange circles, and phosphorylation is labeled as “P” Reproduced and adapted with permission from ref (292). Copyright 2017 American Chemical Society.

Proteoforms of whole protein complexes, correlating PTMs on one subunit to those on another, can be studied by combining native-MS ionization of whole complexes (see section 3.4.2) with top-down sequencing. Skinner et al. developed a native proteomics workflow to identify 217 proteoforms, including those of complexes such as dimeric creatine kinase M or DJ-1. Hybrid native and proteomic techniques were applied to identify the proteoforms of the C8 complex directly from human plasma.^291^ In our lab, a multistage native MS strategy was used to examine how the endogenous yeast FBP1 complex adapts to glucose-rich conditions and heat shock^292^ (see Figure 7). This study revealed that the complex responds differently to changes in growth conditions by tuning phosphorylation dynamics, thus illuminating the role of this PTM in FBP1. In another study, we used a top-down native approach to identify a new proteoform of the 20S proteasome PSMA7 subunit, a proteoform which would have eluded detection by bottom-up proteomics due to its proximity to a lysine-rich region subject to tryptic digestion.^293^

#### 3.4.2 Native MS of Protein Complexes

##### 3.4.2.1 Overview of the Method

Native mass spectrometry takes the top-down approach one step further by retaining aspects of the protein’s native tertiary and quaternary structure in the gas phase.^294^ In native MS, conditions are optimized to ionize protein complexes while retaining noncovalent interactions, with experimental evidence confirming that aspects of native complex structure are maintained in the gas phase.^295^ Complexes can then be broken down in the gas phase by tandem-MS techniques. Native-MS can have increased signal-to-noise relative to denatured top-down methods because folded proteins populate fewer charge state peaks, increasing the intensity of the remaining peaks.^296,297^

The addition of IM (see section 3.2.4) to the native-MS workflow yields information on the 3D shape of protein complexes.^135^ IM separates ions according to their collision cross section, which reflects complex conformation and mass, providing a source of structural information.^126−128^ This data can be combined with modeling tools that use collision cross sections to refine and develop structural models^298,299^ (see section 3.5).

Native MS studies of protein complexes directly measure both composition and stoichiometry of protein complexes because quaternary structure is preserved in the gas phase (see Figure 4B for an example). Moreover, different coexisting complexes are separated in the spectrum based on their masses and can be compared in terms of their relative abundances for a particular sample preparation. Tandem-MS dissociates the complexes into their components to assign and confirm complex composition, to distinguish between core and peripheral subunits and determine subunit network. Contaminants are also easily distinguished because they are not part of the complex charge series.

Analyzing native-MS spectra involves assigning different peaks in the spectra to charge state series for proteins or complexes of interest. This is can be done manually or via computational spectral deconvolution. A variety of softwares exist to enable this, including open source softwares such as UniDec, which deconvolutes spectra using Bayesian inference,^300,301^ NaVia, a program to augment manual assignment,^302^ as well as vendor-provided software. Software for analyzing native IM-MS experiments presents several challenges, as reviewed in ref (299). Data must be interpreted to assign and extrapolate charge states as well as determine collision cross sections, which can then be used to develop structural models. Determining the collision cross section is typically done via a standard calibration curve,^303^ with an open source automated collision cross section software recently introduced by Metz and co-workers.^304^ Software has also been introduced to analyze IM experiments coupled to gas-phase activation or collision induced unfolding experiments.^305,306^

The main drawback of native-MS for determining protein complex composition is that relatively large quantities of complexes are needed for each experiment, with at least 10 μL of midnanomolar concentration required for a detailed native-MS experiment. Assignment of native MS peaks to particular complexes also requires some preknowledge of expected molecular weights in the sample. Therefore, it is useful to have preknowledge of the proteins present in solution and the exact molecular weights of the different subunits, information that can be obtained from proteomics and intact MS of denatured samples, respectively (see section 3.4).

Recent advances in native MS have the potential to extend its applicability to endogenous complexes available in limited quantities. One example is the development of individual ion MS via charge detection mass spectrometry,^307^ in which the ion charge is measured directly to generate a mass spectrum rather than a spectrum showing mass-to-charge ratio. Implemented on various experimental platforms including Orbitrap^308^ and ion trap FT instruments,^309^ this method has provided isotope resolved spectra of complexes as large as β-galactosidase, 466 kDa. This can enable very high resolution spectra of endogenous assemblies in complex mixtures because the measurement of an ion’s true mass resolves ambiguities in crowded spectra.

##### 3.4.2.2 Application to Endogenous Complexes Analysis

Endogenous affinity purification followed by native-MS has been used by multiple laboratories to characterize the yeast exosome.^310−313^ This work has determined both the stoichiometry of various exosome components as well as compositional differences between nuclear and cytoplasmic exosome complexes. Synowsky and Heck showed via native-MS that the Ski complex, which associates with the yeast exosome, is a heterotetramer as opposed to a heterodimer as previously assumed.^314^ Novel complexes have also been directly identified via native-MS; using endogenous purification from muscle tissue followed by native-MS, we identified novel complexes consisting of Arp2/3 subunits and vinculin, likely involved in the formation of focal adhesion complexes^18^ (Figure 4B). New subunits for existing complexes can also be seen in native-MS spectra. For example, COP9 signalosome (CSN) complexes purified directly from erythrocytes harbored a novel subunit, named CSNAP, that had previously evaded detection.^17^ Semi-high throughput native proteomics workflows, analogous to the fractionation-MS bottom-up methods described above, have also been developed.^19,25^ Intriguingly, Skinner et al.^19^ detected novel homomers, which often elude bottom-up proteomic approaches that rely on indirect measurement of protein association.

In addition to soluble complexes, native MS can be applied to membrane proteins, work pioneered by Robinson and co-workers.^315^ By ejecting endogenous membrane complexes directly from native membranes,^316^ complexes of the chaperone DnaK and the outer membrane protein OmpA were observed, confirming the involvement of DnaK in membrane porin assembly. Endogenous membrane proteins can also be purified, and Gross and co-workers studied endogenous photosynthetic reaction centers via native-MS in detergent micelles.^317^

Moreover, the method can be applied to very large protein complexes such as the ribosome (2.30 mDa) and its subcomplexes. Native and tandem-MS of the ribosome stalk complexes from a wide range of organisms revealed differences in stalk composition and stoichiometry.^318^ Intact protein MS of ribosomes examined the stoichiometry of different ribosomal proteins, revealing heterogeneity and substoichiometric binding of ribosome associated proteins.^319,320^ Affinity captured NPC endogenous complexes, which are upward of 80 megadaltons, were analyzed by native MS, confirming the overall stoichiometry and membrane components of the NPC complex.^184^

Because protein complexes retain their solution noncovalent interactions, complex topology can be determined by analyzing the pattern of subunit dissociation in tandem native MS experiments (Figure 5B). Generation of subcomplexes can be aided by addition of small amounts of methanol or other solvents that disrupt hydrophobic interactions, thus encouraging dissociation across weaker quaternary interfaces. Subunit connectivity is then determined by assuming that when large complexes dissociate, they generate subcomplexes from proteins already proximal in the intact structure. This method was used to determine the topologies of a range of endogenous yeast complexes,^311−314,321^ including structural models of the yeast exosome that were later confirmed by crystallographic studies.^311,321^

As discussed above, IM can also be used to derive structural information on endogenous complexes. While IM has not been broadly applied to purified endogenous complexes, our lab recently characterized orthologous endogenous 20S proteasome complexes from a range of species using IM, demonstrating an enlargement of the complex size from the archaeal prokaryotic complex to the eukaryotic 20S proteasomes in yeast and mammals.^293^

Protein complexes are often modulated by cofactor binding. Native mass spectrometry is uniquely suited to the analysis of cofactor/complex binding because cofactors will appear as mass shifts in the MS spectrum of the complex. Moreover, a single native-MS experiment can reveal the full range of coexisting complexes, allowing relative ratios to be quantified in a single experiment.^322^ Skinner et al. in their top-down proteomic study^19^ demonstrated that the holoenzyme of α-enolase was only present in the dimer, as opposed to the monomer, indicating that Mg stabilizes the dimer, and that GDP and magnesium supported formation of the RHOA heterodimers with RHOGDI1. In a similar study from *E. coli*, Shen and co-workers^25^ identified protein complexes bound to metals and glutamine.

**Figure 8:**
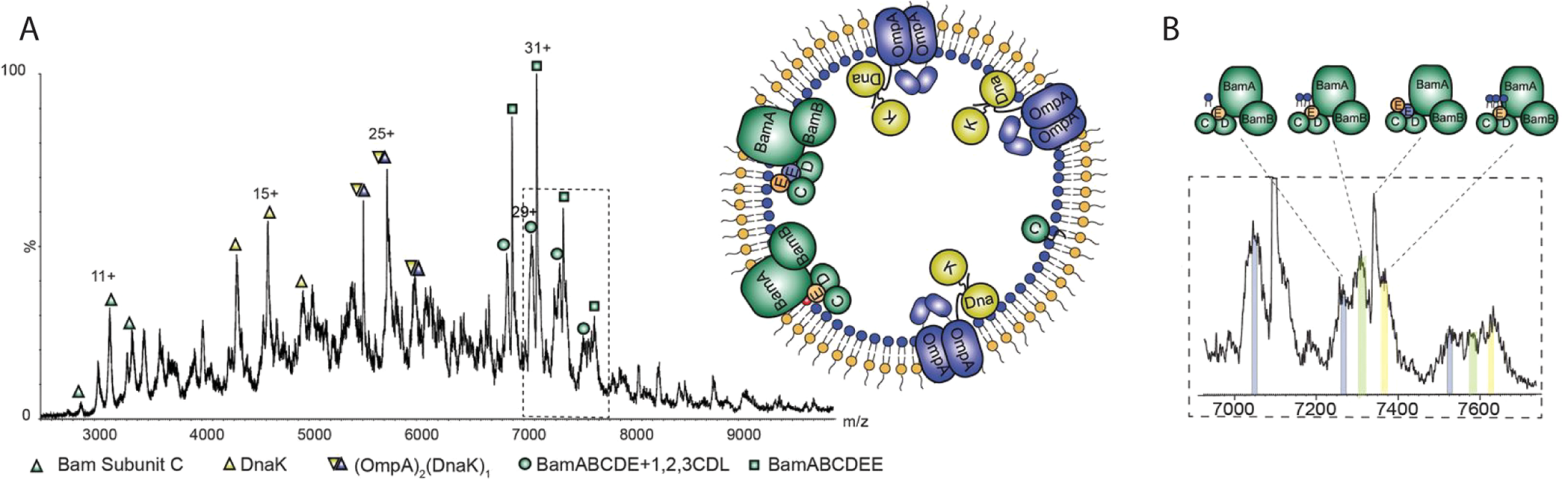
Native-MS spectra show lipid binding to endogenous membrane proteins. (A) Full spectrum of protein complexes observed from the *E. coli* outer membrane. The inset depicts observed complexes of an outer membrane vesicle. (B) Expansion of the mass spectrum assigned to the Bam complex (boxed region in (A)), with monomeric BamE binding to one, two, and three cardiolipins (gray, green, and yellow, respectively). Native-MS thus uncovers the range of lipid bound complexes present in the sample. Reproduced with permission from ref (316). Copyright 2018 The American Association for the Advancement of Science.

The impact of biomolecule binding can be also studied in the context of membrane protein complexes. Robinson and co-workers have made seminal contributions to the use of mass spectrometry to study the binding of lipids to protein complexes, many of them endogenously isolated from host membranes^242,316^ (Figure 8). Membrane proteins display preferences for which endogenous lipids they bind,^323^ and special tandem MS methodologies enable the identification of lipids directly from samples of purified membrane proteins.^324^ These experiments have revealed the stoichiometry of lipid-membrane protein binding^242^ and investigated the effect of lipids on membrane protein stability.^325^ For example, in complexes ejected directly from endogenous membranes, the BAM complex from *E. coli* membranes was observed bound specifically to cardiolipin (Figure 8), while ANT-1 from the mitochondrial membrane bound fatty acids with palmitate headgroups, among others.^316^ These studies are often complementary to high-resolution cryo-EM investigations of membrane proteins, where the identity of the bound lipid is unknown.

Native MS can also be accomplished directly from crude samples, detecting abundant proteins without the need for purification.^326^ This approach uses the limited dynamic range of MS as an advantage, and clear spectra can be acquired of abundant proteins. In an extension of the direct-MS approach, native MS was recently applied to individual erythrocytes yielding spectra of hemoglobin, which constitutes the vast majority of protein in erthrocytes.^327^ Individual cells were selected and a microinjector used to transfer the cells into an MS emitter. However, these methods are limited to very abundant proteins; to apply this technique to a broader range of proteins, online separation and increased signal-to-noise must be applied.

### 3.5 Integrative Approaches and Computational Modeling

As described above, each MS method provides specific information about endogenous complexes, and each has their own pros and cons. The information provided from different MS experiments is complementary; for example, bottom-up proteomics can be exquisitely sensitive to complex components while native-MS directly detects whole complexes. Therefore, it is often advantageous to combine multiple MS modalities to derive a more complete functional and structural model of a particular endogenous complex. Termed “hybrid” mass spectrometric approaches,^230,320^ in this method, information from different experimental modalities is combined to characterize complexes. For example, Robinson, Aebersold, and co-workers combined information from native-MS, bottom-up proteomics, chemical cross-linking, and IM to derive integrative models of the 26S proteasome lid complex and its submodules.^230^ Similarly, Heck and co-workers used hybrid MS approaches to analyze a ribosomes^320^ from multiple kingdoms of life. For native MS experiments that determine complex stoichiometry, bottom-up proteomics are often important to identify the composing subunits,^311,314^ with complex stoichiometry determined by native-MS measurement of the intact complex.

Moreover, information from MS can be used in combination with computational molecular modeling tools, with MS data serving as a restraint for the generation of complete structural models. Cross-linking MS is a natural companion to computational modeling, as described in ref (223). Chemical labeling methodologies can also be combined with computational methods to determine protein structures and topographies. Huang et al. advanced the field by converting hydroxyl radical footprinting data into a protection factor that can be used as a restraint in molecular modeling.^328^ Similarly, hydroxyl radical footprinting data has been used to differentiate between molecular models^329^ and combined with molecular dynamics data for model determination data.^330^ IM can also be combined with modeling tools that use the collision cross section as a restraint to refine and develop structural models.^298,299,331^ Because of the sensitivity and robustness of these methods, they hold great promise for the study of endogenous complexes available in limited quantity.

Moreover, modeling tools form the basis of integrative structural biology,^333^ of which MS is often an important component. As described in refs (333 and 334), this experimental approach combines data from multiple experimental and theoretical methods to build a model of a large biological system for which no single method is sufficient. A wide range of experimental data can be used, including different MS modalities, cryo-EM, SAXS, FRET, and protein–protein interaction data. Different types of experimental and theoretical data are phrased as restraints, and an algorithm driven by a scoring function determines the structural and dynamic model that satisfies most data. Central to this experimental approach is flexible software that can generate the complete range of structures that satisfy the available data. Popular available programs include Rosetta^335^ and the Integrative Modeling Platform (IMP),^336^ although there are other options available that may be tailored to a particular experimental tool, as described in refs (334 and 337).

Several important and extremely large structural models have been built using integrative tools that use MS data as a central modality. For example, MS data has played an integral role in the structural biology of the nuclear pore complex. One of the first integrative models was reported in 2007 by Alber and co-workers,^338^ later updated in 2018,^184^ combining information from proteomic affinity experiments, charge detection native-MS, cross-linking MS, cryo-EM, among other information, to develop a full structural model of the complete NPC, which is greater than 40 mDa. Beck and co-workers also used integrative modeling, relying again on cross-linking MS and cryo-EM tomography, to analyze the full nuclear pore complex^339^ and build a model of the inner scaffold ring.^340^ Cross-linking MS along with cryo-EM data was used to describe the architecture of the 10 mega Da pyruvate dehydrogenase supercomplex.^332^ Other examples of structural models relying on MS data include the 26S proteasome,^231^ constructed by combining cross-linking MS, proteomics interaction data, and cryo-EM, and the human Polycomb repressive complex 2 complex, generated primarily by cryo-EM but also with cross-linking MS data.^341^

Another related area is the increasing use of MS for structural proteomics efforts that combine MS and cryo-EM for the analysis of cryo-EM grids prepared from crude fractions as reviewed in ref (342). MS identification of components in SEC or sucrose gradient fractions is key to enabling the fitting of electron density maps to specific proteins.^343−345^ Integrating the MS methods described here with these and other emerging structural biology techniques will only further the study of endogenous complexes.

## 4 Conclusion and Future Directions

Biological MS has made seminal contributions to the analysis of endogenous protein complexes. Depending on the technology chosen, insights range from characterizing the interaction partners of a specific protein to determining the topology of endogenous complexes to analyzing conformational changes between different states. Importantly, by determining these properties for endogenously isolated protein complexes, results are generated that report on biologically relevant interactions and conformations.

Over the course of this review, it has been repeatedly highlighted that one of the main challenges in characterizing endogenous complexes is their low abundance, which is in part what makes MS a useful tool for characterizing such complexes. One of the main solutions to this issue is to process larger amounts of starting material. While it is relatively straightforward to scale up yeast and bacterial growth protocols, even in an academic setting, it is more difficult and costly for eukaryotic cells. HEK293 cells and other strains have been adapted to growth in high density suspension culture but may not be the most relevant physiologically due to altered gene expression.^346^ Importantly, for native-MS, emerging research indicates that enrichment, rather than complete purification, can yield well-resolved spectra of proteins.^326^ Thus, for native MS efforts where intact complexes are detected, proteins may not need to be fully purified for complex identification.

Advances in mass spectrometer technology are also increasing the sensitivity of measurements and enabling application of biological MS to less abundant proteins. Single-molecule MS,^347^ in which both charge and *m*/*z* ratio are measured to directly detect the mass of the ion, was recently applied to native macromolecular protein assemblies on Orbitrap instruments^307,348^ by two different groups. Single-particle charge detection not only increases the sensitivity of native-MS but also permits the resolution of overlapped charge states in heterogeneous samples. Very small amounts of sample can be analyzed with novel microfluidic devices.^349^ For bottom-up proteomics, new instruments that includes variants of IM devices can greatly increase sensitivity and sequencing speed^146^ and were recently shown to identify proteins from single cells.^350,351^ Applying these technologies to the analysis of protein complexes will require miniaturization of purification techniques but can potentially enable comparison of complexes across different tissues available in limited amounts.

This latter goal, namely understanding how complexes vary between tissues and physiological states, is a crucial frontier for biological MS. Thus far, however, characterization of endogenous complexes has largely been limited to model organisms such as yeast and model cell lines such as HEK and HeLa. Extending this approach to the analysis of protein complexes and proteoforms from a range of tissues can be enabled by CRISPR genome tagging approaches, which permit affinity labeling of proteins from a broader range of germ lines. It is also conceivable to construct affinity tagged mice and purify and compare complexes from a broad range of tissues. We expect that in the future biological mass spectrometry will be applied to a broader range of organisms and under healthy and disease states.

It is our hope that this review, while broad and general in its outlook, will serve as a guide to scientists interested in studying endogenous protein complexes and will stimulate the application of biological MS to a broader range of problems.
